# Dual-ligand curcin-loaded hybrid solid lipid nanoparticles achieve durable gliosarcoma remission while preserving neuro-behavioral function

**DOI:** 10.7150/thno.123534

**Published:** 2026-03-04

**Authors:** Mohamed Sheikh Mohamed, Srivani Veeranarayanan, Yasushi Sakamoto, Rie Suge, Narumi Hirosawa, Aby Cheruvathoor Poulose, Toru Mizuki, Toru Maekawa

**Affiliations:** 1 Bio-Nano Electronics Research Centre, Toyo University, 2100 Kujirai, Kawagoe, Saitama, 350-8585, Japan.; 2 Graduate School of Interdisciplinary New Science, Toyo University, 2100 Kujirai, Kawagoe, Saitama, 350-8585, Japan.; 3 Division of Bacteriology, Department of Infection & Immunity, Jichi Medical University, Shimotsuke-Shi, Tochigi, 329-0498, Japan.; 4 Biomedical Research Centre, Division of Analytical Science, Saitama Medical University, Saitama 350-0495, Japan.; 5 Department of Liberal Arts, Saitama Medical University, Saitama 350-0495, Japan.; 6 Regional Centre of Advanced Technologies and Materials, Czech Advanced Technology and Research Institute (CATRIN), Palacký University in Olomouc, Šlechtitelů 27, 783 71, Olomouc, Czech Republic.

**Keywords:** gliosarcoma, solid lipid nanoparticles, transferrin, RGD, anti-cancer therapy, ribosome inactivating proteins

## Abstract

**Rationale:**

Gliosarcoma (GSM) is a rare, highly invasive glioblastoma subtype with limited therapeutic options and a poor prognosis. We report the first dual-ligand hybrid solid lipid nanoparticle (HSLN) system for blood-brain barrier (BBB) penetration and targeted delivery of curcin, a type I ribosome-inactivating protein (RIP), to orthotopic GSM.

**Methods:**

HSLNs (~150-200 nm, polydispersity index (PDI) < 0.3, zeta potential (ζ) ≈ -8 mV) were co-functionalized with transferrin and RGD peptides at optimized 4:6 ratios to enhance BBB transcytosis and tumor uptake. Physicochemical, cytotoxicity, and docking studies assessed curcin stability, bioactivity, and multi-receptor interactions. In vivo biodistribution, proteomics, and therapeutic efficacy were evaluated in BALB/c-nu and ICR-nu orthotopic GSM models, with neurobehavioral assessments for functional preservation.

**Results:**

Intravenous curcin-loaded dual-ligand HSLNs achieved complete tumor regression in 60% of BALB/c-nu and 90% of ICR-nu mice, extending median survival from 14 to 38 days in BALB/c-nu mice and resulting in median survival not reached in an independent ICR-nu cohort. Neurobehavioral function was preserved during treatment. Biodistribution and proteomic analyses confirmed efficient BBB penetration, tumor-selective accumulation, and suppression of VEGFA/C, MMP-9, PDGFB, and SERPINE1. Molecular docking revealed strong binding of curcin to GSM-associated receptors (EGFR, EphA2, mGluR6, and IL-13Rα2).

**Conclusions:**

Stoichiometry-optimized dual-ligand HSLNs enable targeted, BBB-penetrant delivery of curcin, achieving durable GSM remission with functional preservation. This theranostic-ready platform combines therapeutic potency with tumor specificity, offering a promising strategy for ribosome-inactivating protein delivery in GSM and other CNS malignancies.

## Introduction

Gliosarcoma (GSM) is a rare and highly aggressive variant of glioblastoma multiforme (GBM), constituting approximately 2-5% of GBM diagnoses 1. Characterized by a biphasic histology comprising both glial and sarcomatous elements, GSM is classified as a World Health Organization (WHO) grade IV isocitrate dehydrogenase (IDH)-wildtype glioma and demonstrates a more invasive clinical trajectory than conventional GBM 2. Despite employing the current GBM standard-of-care, maximal surgical resection followed by radiotherapy and temozolomide (TMZ) chemotherapy, GSM patients face a median survival of only 8.3-11.5 months, which is substantially shorter than in GBM 3. Additionally, GSM exhibits a greater propensity for extracranial metastasis, including to the lungs, liver, and lymph nodes, complicating clinical management and contributing to poor prognosis 4.

One of the principal therapeutic challenges in GSM is its profound intratumoral heterogeneity, stemming from both its dual histological nature and a complex tumor microenvironment (TME). The TME comprises glioma stem-like cells, tumor-associated macrophages, stromal fibroblasts, and immunosuppressive mediators, all of which facilitate tumor growth, angiogenesis, and resistance to therapy 5. Compounding this challenge is the blood-brain barrier (BBB), a selective endothelial interface that restricts the passage of therapeutic agents. Even when partially compromised and transformed into a blood-tumor barrier (BTB), its permeability remains inconsistent, thereby limiting drug delivery and increasing the risk of systemic toxicity 6.

Most GSM treatments are extrapolated from GBM clinical protocols, despite growing evidence that molecular and phenotypic differences may render such approaches suboptimal 1. Notably, TMZ, the standard chemotherapeutic agent for GBM, exhibits reduced efficacy in GSM, potentially due to high O6-methylguanine-DNA methyltransferase (MGMT) expression and alternative DNA repair mechanisms, which are well-characterised mediators of TMZ resistance in GBM 7. Given the frequent MGMT upregulation observed in GSM 1, it is reasonable to hypothesize that a similar, if not enhanced, resistance mechanism may contribute to the chemoresistant phenotype of GSM, underscoring the need for distinct therapeutic strategies. Furthermore, therapies like tumor-treating fields (TTFs) 8 and immune checkpoint inhibitors, while gaining ground in GBM, have yet to show significant benefit in GSM and remain investigational 9, 10. Additionally, while aggressive multimodal regimens, including surgery, radiotherapy, and chemotherapy, are often employed in pediatric gliomas to achieve maximal tumor control, such approaches frequently result in significant neurotoxicity and long-term cognitive sequelae, particularly in younger patients whose developing brains are more susceptible to treatment-related injury 11, 12. Despite its clinical severity, GSM remains significantly underexplored compared to GBM. Even comprehensive genomic studies often pool GSM cases within GBM cohorts, limiting subtype-specific insights and hindering the development of tailored therapeutic strategies 13. The scarcity of dedicated clinical trials and the reliance on retrospective data sets further hinder progress in identifying GSM-specific therapeutic strategies.

Among emerging alternatives, nanoparticle-based drug delivery systems (NP-DDS) offer a promising approach for overcoming BBB limitations and enhancing tumor selectivity via the enhanced permeability and retention (EPR) effect 14. However, despite advances in this field, clinical translation to central nervous system (CNS) malignancies remains limited by persistent challenges such as suboptimal BBB penetration, rapid systemic clearance, and the variable efficacy of tumor-targeted strategies. To address these limitations and enhance therapeutic localization, receptor-mediated transcytosis has emerged as a promising approach, whereby nanoparticles are functionalized with ligands that recognize receptors overexpressed on both BBB endothelial cells and glioma tissues 15, 16. In this context, transferrin receptors (TfR) and integrins, particularly αvβ3 and α5β1, have emerged as two of the most promising molecular targets for nanoparticle-mediated drug delivery to gliomas. Arginine-glycine-aspartic acid (RGD) peptides, which specifically bind to integrins overexpressed on tumor vasculature and glioma cells, have demonstrated enhanced intratumoral penetration and cellular uptake in various glioma models 17, 18. Similarly, transferrin-conjugated platforms exploit the overexpression of TfRs on both brain capillary endothelial cells and glioma cells, enabling dual functionality: enhanced BBB transcytosis and tumor-specific targeting 19, 20. Recent advancements in dual-targeting strategies that combine both transferrin and RGD ligands on a single nanoparticle construct have demonstrated additive or even synergistic effects on BBB traversal, glioma cell binding, and therapeutic payload accumulation. These multifunctional systems offer superior targeting efficiency and drug bioavailability compared to monovalent approaches, positioning them as a next-generation platform for brain tumor therapy 21, 22.

Simultaneously, ribosome-inactivating proteins (RIPs) have emerged as a novel class of therapeutic agents with the ability to irreversibly halt protein synthesis by depurinating 28S rRNA, thereby rendering ribosomes catalytically inactive 23, 24. Among these, curcin, a type I RIP derived from *Jatropha curcas*, has demonstrated potent cytotoxicity against glioma cells under *in vitro* conditions 20, 25, 26. Unlike conventional chemotherapeutics such as TMZ or carmustine (BCNU), which rely on DNA alkylation and often induce resistance through MGMT expression or mismatch repair deficiencies, curcin operates via a distinct mechanism 25, 27-29, making it an attractive alternative for resistant glioma subtypes. Given the frequent activation of DNA repair mechanisms and the limited efficacy of DNA-alkylating agents in GSM, therapeutic strategies that act independently of DNA damage are highly desirable. RIPs such as curcin fulfill this requirement by irreversibly inhibiting protein synthesis via 28S rRNA depurination, a mechanism orthogonal to MGMT-mediated resistance and well suited for chemoresistant GSM. However, curcin's clinical translation has been hindered by poor membrane permeability, short systemic half-life, and susceptibility to proteolytic degradation 23, 27.

To overcome these limitations, we developed a dual-targeted hybrid solid lipid nanoparticle (HSLN) formulation encapsulating curcin and surface-functionalized with both transferrin and RGD. Solid lipid nanoparticles offer advantages such as controlled release, high biocompatibility, and tunable surface chemistry, making them ideal carriers for macromolecules like curcin 26, 30, 31. The dual-ligand configuration is designed to exploit complementary transcytotic pathways across the BBB and enable selective tumor accumulation, while the encapsulation protects curcin from enzymatic degradation and facilitates intracellular delivery.

GSM is recognized as a distinct clinicopathological entity; however, several barriers to effective treatment, including restricted BBB transport and limited therapeutic penetration, overlap with those observed in other high-grade gliomas. These shared constraints highlight the need for delivery strategies capable of achieving effective intracranial drug exposure in GSM. This study presents one of the first preclinical applications of a dual-ligand, curcin-loaded nanoparticle platform specifically designed for GSM therapy. The formulation is tailored to overcome the three central barriers in GSM treatment: limited BBB penetration, intratumoral heterogeneity, and therapeutic resistance. The HSLN-curcin system represents not only a promising precision therapy for GSM but also a modular platform adaptable to other CNS malignancies through ligand and payload customization.

## Materials and Methods

### HSLN preparation and characterization

#### Preparation of curcin-loaded, void, and CdSe QD-loaded HSLNs

HSLNs composed of DSPE-PEG(2000) Amine [1,2-distearoyl-*sn*-glycero-3-phosphoethanolamine-N-[amino(polyethylene glycol)-2000] (ammonium salt) (Avanti Polar Lipids, USA), stearic acid, and lecithin (both from Sigma-Aldrich, USA) were synthesized via a modified lipid co-acervation method 26. Each lipid component (100 mg) was dissolved in 5 mL of chloroform and desiccated overnight to form a thin film. For drug loading, the film was hydrated with 2 mL phosphate-buffered saline (PBS) containing 16 mg of purified curcin (isolated per previously published protocol 25). Mild sonication (45 kHz, 1 min) produced a stable dispersion of non-targeted (NT) HSLNs. Void HSLNs were prepared identically using drug-free PBS. Ultracentrifugation (50,000 rpm, 30 min) resulted in an overall nanoparticle recovery of approximately 60-65%, calculated based on the dry mass of the pelleted nanoparticles relative to the total input formulation. Comparable recovery was obtained for both void and curcin-loaded HSLNs, indicating that curcin encapsulation did not adversely affect particle integrity or purification efficiency. The resulting nanoparticle pellets were washed with PBS and stored at 4 °C until further use.

CdSe quantum dots (QDs) were synthesized as previously described 32, suspended in chloroform (2 mg), and co-loaded during lipid film formation. The QD-lipid films were hydrated and processed identically to obtain QD-loaded HSLNs.

#### Preparation of targeted HSLNs

Surface decoration of HSLNs was carried out by carbodiimide coupling. Human transferrin and RGD peptide (Arg-Gly-Asp) (both Sigma-Aldrich, USA) were dissolved in ice-cold 0.1 M 2-(N-morpholino)ethanesulfonic acid (MES) buffer (pH 5.5) and activated for 15 min with 10 mM 1-ethyl-3-(3-dimethylaminopropyl)carbodiimide (EDC) and 10 mM N-hydroxysuccinimide (NHS) (equimolar to ligand carboxyl groups; Tokyo Chemical Industry, Japan) to generate NHS esters. The activated ligands were transferred without delay to 10 mL of HSLNs (100 mg lipid equivalent) dispersed in PBS (pH 7.2) and tumbled overnight at 4 °C. To provide a 1.5-fold molar excess over the estimated number of surface amines, thus buffering against NHS-ester hydrolysis while avoiding reagent waste, 2.0 mg NHS-transferrin (TF) (≈ 25 nmol) was added for TF-only conjugation, and 0.09 mg NHS-RGD (≈ 150 nmol) for RGD-only conjugation. Dual-ligand particles were prepared by premixing NHS-TF and NHS-RGD at the desired molar ratios (RGD₂\:TF₈, RGD₄\:TF₆, RGD₆\:TF₄, RGD₈\:TF₂) while maintaining the same overall ligand-to-nanoparticle mass ratio. After the overnight reaction the suspensions were diluted with PBS and centrifuged at 50, 000 rpm for 30 min at 4 °C. Pellets were washed twice with PBS and subsequently stored at 4 °C. Unbound TF in the combined supernatants was quantified with the Pierce Micro-BCA Protein Assay Kit (Thermo Fisher Scientific), and free RGD was determined with fluorescamine reagent (Sigma-Aldrich) according to the manufacturers' protocols. Ligand-coupling efficiency was calculated as (ligand fed - ligand recovered) / ligand fed × 100%. To assess formulation reproducibility, targeted HSLNs were synthesized repeatedly over the course of the study (~10-15 independent batches). Ligand conjugation efficiency and surface density were quantitatively evaluated in five representative batches. TF and RGD coupling efficiencies remained consistent across batches, with variability typically within ± 5-8% of the target stoichiometry, demonstrating minimal batch-to-batch variation and good reproducibility of the dual-ligand functionalization process.

#### Particle characterization

Morphological analysis was performed by transmission electron microscopy (TEM; JEM-2100, JEOL, Japan) using 20 μL samples drop-cast on carbon-coated, hydrophilized copper grids. Size measurements were manually recorded for 100 individual particles. For elemental mapping in HSLN-CdSe, scanning TEM (STEM) with energy-dispersive X-ray spectroscopy (EDS; JEOL) was used. X-ray photoelectron spectroscopy (XPS) was performed using a HAXPES system (ULVAC-PHI, Japan), and photoluminescence spectra were acquired using a spectrofluorometer (FP-750, JASCO, Japan).

Dynamic light scattering (DLS) and zeta potential (ζ) were assessed using Zetasizer Nano ZS (Malvern Panalytical, UK). sodium dodecyl sulfate-polyacrylamide gel electrophoresis (SDS-PAGE) was used as an orthogonal method to visualise TF coupling. The respective samples (TF, TF-HSLNs, Dual-HSLNs) were resolved on a 10% polyacrylamide gel; the 79 kDa band was quantified by densitometry (ImageJ 1.52a, NIH, USA) against a five-point TF standard curve. In parallel, the unreacted protein present in the final wash fractions was measured with the Pierce Micro-BCA Protein Assay Kit using a standard protein calibration; conjugation efficiency was calculated from the difference between the mass of TF fed and the mass recovered. For RGD, the residual peptide in the combined supernatants was quantified with fluorescamine reagent. An RGD standard curve was prepared, covering the expected 0.09 mg feed and allowing direct calculation of the unbound fraction. Covalent attachment on the nanoparticle surface was further confirmed by matrix-assisted laser desorption/ionisation time-of-flight mass spectrometry (MALDI-TOF MS; AXIMA-CFR, Kratos Analytical, UK) using α-cyano-4-hydroxycinnamic acid (CHCA) matrix with detection of the characteristic 588.35 m/z peak. Ligand density (molecules particle⁻¹) was obtained by converting the bound mass determined from the Micro-BCA (TF) or fluorescamine (RGD) assays into moles, correlating those values with the band or peak intensities from SDS-PAGE or MALDI-TOF standard curves, and normalising to the total nanoparticle concentration calculated from the dry mass of the formulation and its DLS-derived mean diameter and density. Formulation stability was tracked by monitoring hydrodynamic size and ζ-potential over six months at 4 °C. To assess protein-corona formation and chemical stability, HSLNs were incubated in 50% FBS (Gibco, USA) for 24 h, and post-incubation size and ζ-potential were recorded to detect surface-charge or colloidal changes.

#### Drug loading and release studies

Encapsulation efficiency (EE%) and drug release were quantified via ultraviolet-visible (UV/Vis) spectrometry (DU730, Beckman Coulter, USA). HSLNs were lysed with 10% Triton X-100 (Sigma-Aldrich), and absorbance was measured against a standard curcin calibration curve. EE was calculated using:


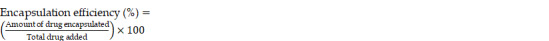

(1)

*In vitro* release studies were conducted in PBS at pH 7.4, 6.5, and 4.0, corresponding to physiological conditions, tumor microenvironment acidity, and late endosomal/lysosomal acidity, respectively. Before release experiments, curcin-loaded HSLNs were purified by pelleting and resuspension to remove unencapsulated curcin. Purified nanoparticles were suspended at a final concentration of 10 mg/mL in PBS (1.5 mL per tube) and incubated at 37 °C in a temperature-controlled incubator (As One, Japan) under gentle continuous tube rotation (10 rpm) using a mini rotator (As One, Japan).

At predetermined time points (1, 6, 12, 24, 48, and 96 h), independent samples were processed (destructive sampling). Nanoparticles were separated by ultracentrifugation (50,000 rpm, 10 min, 25 °C), and the supernatant (soluble fraction) was collected for analysis. Curcin concentration in the supernatant was quantified using a curcin calibration curve at 272 nm (220 nm used as a supportive readout), using buffer-only and blank HSLN controls for baseline correction. Cumulative drug release was calculated as follows:


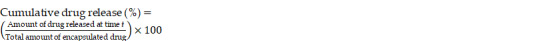

(2)

All *in vitro* experiments were performed in triplicate, and where applicable, results reflect consistent nanoparticle batches with minimal batch-to-batch variability.

### *In vitro* studies

#### Cell culture

Human cortical neurons (HCN-1A) and astrocytes (HA) were obtained from the American Type Culture Collection (ATCC, USA). Human brain pericytes (BPC) and brain endothelial cells (BEC) were purchased from Cell Systems (USA). U-87MG were kindly provided by Prof. Kazuhiko Mishima, Saitama Medical University, Japan. GI-1 cells (RCB0763; RIKEN Bioresource Center, Japan) were originally established from a WHO Grade IV human gliosarcoma surgical specimen 33, 34 and used to generate gliosarcoma xenografts. For *in vivo* studies, 5 × 10^5^ GI-1 cells in 2 µL PBS were stereotactically implanted into the right striatum of athymic nude mice to generate GSM xenografts. HCN-1A, HA, and GI-1 cells were cultured in Dulbecco's Modified Eagle's Medium (DMEM; Gibco, USA) supplemented with 10% fetal bovine serum (FBS) and 1% penicillin-streptomycin (Gibco, USA). BPC and BEC were maintained in complete Classic Medium (Cell Systems, USA). All cells were incubated at 37 °C in a humidified atmosphere with 5% CO₂. *All in vitro experiments were conducted in triplicate.*

#### Cell viability assay

HCN-1A, HA, BEC, BPC, and GI-1 cells were seeded in 96-well plates at 1 × 10³ cells/well. After 48 h, cells were treated with curcin (0-100 μg/mL, diluted in growth medium) or respective controls. For nanoparticle-based studies, void HSLNs, HSLN-CdSe, and curcin-loaded formulations (NT, RGD, TF, Dual) were added at concentrations equivalent to 0-500 μg/mL or 30 μg/mL of encapsulated curcin, as appropriate. After 72 h, cell viability was measured using the Alamar Blue assay (Invitrogen, USA). Emission was recorded at 590 nm (excitation 520 nm) using a PowerScan HT microplate spectrophotometer (Dainippon Sumitomo Pharma, Japan). Viability was expressed as a percentage of untreated control wells. Half-maximal inhibitory concentration (IC₅₀) values were determined by nonlinear regression analysis of dose-response curves.

#### *In vitro* BBB model construction

**Model 1**: Transwell inserts (Iwaki, Japan) were coated with ~25,000 BPC on the abluminal surface (lower side) and incubated for 24 h in an inverted position. Subsequently, ~25,000 BECs were seeded on the luminal side (upper surface). The constructs were maintained until transendothelial electrical resistance (TEER) exceeded 300 Ω·cm². Blank inserts served as negative controls. **Model 2**: GI-1 cells (~25,000) were seeded in the bottom wells of a 24-well plate before placing the Model 1 insert on top to mimic a tumor-facing BBB configuration.

#### BBB integrity and permeability analysis

BBB tightness was validated by medium permeation and apical-to-basal permeability studies. In the first assay, 0.5 mL of culture medium was added to the transwell upper chamber, and volume permeation into the lower chamber after 30 min was quantified. Lower permeation volumes indicated tighter junctions. In the second assay, fluorescein isothiocyanate (FITC)-inulin, FITC-dextran 20 kDa, and FITC-dextran 70 kDa (Sigma-Aldrich, USA) were added to the apical side, and fluorescence in the basolateral compartment was measured after 15 min using a multimode spectrofluorometer (Dainippon Sumitomo Pharma, Japan).

#### BBB penetration of curcin-FITC and HSLNs

Curcin (1 mg, ~36 nmol, MW ~28 kDa) was dissolved in 0.1 M sodium carbonate buffer (pH 9.0). FITC (Sigma-Aldrich, USA; 0.1 mg, ~257 nmol) was dissolved in dimethyl sulfoxide (DMSO) and added dropwise at an approximately 7:1 molar excess relative to curcin. The mixture was incubated at 37 °C for 30 min in the dark with gentle stirring to allow NHS-ester coupling (Dojindo, Japan). Excess FITC was removed by centrifugal filtration (10 kDa MWCO), and the FITC-curcin conjugate was stored protected from light. FITC-curcin or CdSe-loaded HSLNs (NT, TF, RGD, and Dual, including ligand ratio variants) were applied to the apical compartment of the transwell insert and incubated for 4 h. FITC fluorescence (curcin) or CdSe emission (HSLNs) in the basolateral compartment was quantified to assess BBB translocation efficiency.

#### Post-BBB anti-cancer efficacy

Using **Model 2**, curcin and curcin-loaded HSLNs were added to the apical compartment and incubated for 4 h. Inserts were then removed, and GI-1 cells were cultured for 72 h. Viability was measured via Alamar Blue assay, and cytotoxicity was expressed relative to untreated controls. This assay functionally validated BBB-crossing efficacy and therapeutic bioavailability. TEER values were monitored both before and after treatment to assess barrier integrity and potential cytotoxic effects on BEC and BPC layers.

#### *In vitro* cellular uptake studies

GI-1 cells were seeded at 1 × 10⁵ cells/well in 12-well plates and allowed to adhere for 24 h. Cells were then treated with FITC-curcin or CdSe-loaded HSLNs (NT, TF, RGD, and Dual with varied ligand ratios) for 2 h. After incubation, cells were washed with PBS, fixed in 4% paraformaldehyde, and analyzed by flow cytometry (FACS; Intellicyt, USA) to quantify intracellular uptake.

#### Receptor expression profiling

To mechanistically validate the contribution of transferrin receptor (TfR) and integrin-mediated pathways to dual-ligand HSLN uptake, receptor expression, blocking, and endocytic inhibition studies were performed *in vitro*. GI-1 and U-87MG glioma cells, HCN-1A, and BECs were cultured under standard conditions (37 °C, 5% CO₂) and harvested at 70-80% confluence. For receptor expression analysis, cells were washed with ice-cold PBS containing 1-2% fetal bovine serum and incubated with fluorophore-conjugated antibodies against TfR (CD71), integrin α5 (CD49e), and integrin β3 (CD61) at manufacturer-recommended dilutions for 30 min at 4 °C in the dark. Corresponding isotype controls were included to define background fluorescence. After washing, cells were analyzed by flow cytometry, and receptor expression was evaluated by histogram overlays and median fluorescence intensity relative to isotype controls.

#### Receptor-blocking validation of ligand-mediated uptake

For receptor-blocking uptake studies, GI-1 and U-87MG cells were seeded on glass-bottom dishes or multiwell plates and allowed to adhere overnight. Cells were pre-incubated for 30-60 min at 37 °C with either anti-TfR blocking antibody, anti-integrin blocking antibody, matched isotype antibody, or vehicle control, followed by exposure to fluorescently labelled HSLNs at an equivalent particle dose under continuous presence of the blocking antibodies. After incubation, cells were thoroughly washed to remove extracellular particles. Uptake was assessed by confocal laser scanning microscopy.

#### Endocytic pathway inhibition of uptake

To further investigate the involvement of specific endocytic pathways, GI-1 and U-87MG cells were pre-treated for 30 min with pharmacological inhibitors before nanoparticle exposure, including chlorpromazine (CPZ) to inhibit clathrin-mediated endocytosis and 5-(N-ethyl-N-isopropyl)amiloride (EIPA) to inhibit macropinocytosis, with vehicle-treated cells serving as controls. In selected experiments, nanoparticle uptake was additionally evaluated at 4 °C to suppress energy-dependent internalization. Fluorescent HSLNs were then added at the same dose as above and incubated under inhibitor-maintained conditions. Following incubation, cells were washed extensively and analyzed by confocal microscopy using identical acquisition settings across all groups. All experiments were performed in triplicate, and uptake data were expressed relative to the corresponding control condition.

### *In vivo* studies

#### Animal models

Male BALB/c-nu and ICR-nu nude mice (4 weeks old, 20  ±  2 g) were obtained from Charles River Laboratories (Japan) and acclimatized under standard housing conditions (14 h light / 10 h dark cycle) with ad libitum access to low-fluorescence food (SG Corporation, Japan) and water (**Scheme S1**). All procedures were approved by the Institutional Animal Care and Use Committee of Saitama Medical University (Approval No. 2179).

Animals were examined at least twice daily. Any mouse that lost > 5 g (~20% of its baseline mass) or displayed advanced neurological distress (e.g., persistent seizure, lateral recumbency, failure to feed), or other humane-endpoint criteria was humanely euthanised by isoflurane overdose, and tissues were collected. In the rare event that an unsupervised death occurred (e.g., during the dark cycle), the carcass was recovered at the next inspection (≤ 30 min from discovery), immediately perfused with saline, and brains plus major organs were harvested, snap-frozen or fixed, and routed to the planned histological, biochemical, or inductively coupled plasma mass spectrometry (ICP-MS) workflows to avoid sample-loss bias.

Each experimental group consisted of 10 mice (n = 10), randomly assigned using a random number generator.

#### Biocompatibility and biodistribution of HSLNs

Mice (BALB/c-nu, tumor-free) received intravenous injections of void HSLNs (0.5 mg/mouse) every other day for 20 days (10 doses). Saline-injected animals served as controls. Body weight was monitored pre- and post-treatment. At the study endpoint, animals were anesthetized, and portal vein blood (~0.5 mL) was collected for serum biochemistry. Markers analyzed included: Liver function: aspartate aminotransferase (AST), alanine aminotransferase (ALT), alkaline phosphatase (ALP), lactate dehydrogenase (LDH), total bilirubin (T-BIL); Renal function: blood urea nitrogen (BUN), creatinine (CRE), uric acid (UA); Others: total protein (TP), albumin (ALB), albumin/globulin (A/G ratio), total cholesterol (T-CHO), creatine kinase (CK), lipase (LIP), Glucose. Mice were perfused intracardially with 250 mL saline, followed by 4% paraformaldehyde. Brains were post-fixed in 4% paraformaldehyde overnight and cryoprotected in 30% sucrose solution at 4 °C until they sank. Brain, heart, lungs, liver, spleen, and kidneys were then embedded in optimal cutting temperature (OCT) compound, cryosectioned (20 μm, Leica CM1900), and stained with hematoxylin and eosin (H&E). Imaging was performed using a Keyence BZ-X700 microscope.

Biodistribution studies were performed using CdSe-loaded HSLNs (HSLN-CdSe). Mice received I.V. injections (0.5 mg/mouse), and fluorescence imaging was conducted at 6 h using Clairvivo OPT (Shimadzu, Japan). Post-mortem organs and blood were digested using nitric acid in a microwave digestion system (Ethos Easy, Milestone), and cadmium (Cd) content was quantified by ICP-MS (iCAP Q, Thermo Fisher Scientific). Organ samples were normalized to tissue weight (μg Cd/g tissue). In parallel, organ homogenates (10× dilution in PBS, centrifuged) were analyzed for photoluminescence using JASCO FP-750. Three mice per group were used for each endpoint (histology, fluorescence imaging, and ICP-MS quantification). Fluorescence imaging and ICP-MS were performed on separate, non-overlapping cohorts drawn from the same treatment pool to minimise animal use in accordance with the 3 R principles of replacement, reduction, and refinement.

#### Antioxidant and immunotoxicity assays

Liver homogenates (10%) were evaluated for oxidative stress markers using standard colorimetric assays: Lipid peroxidation (LPO): expressed as nmol/mg protein; Glutathione reductase (GR) and glutathione peroxidase (GPX): nicotinamide adenine dinucleotide phosphate (NADPH)-dependent reduction; Reduced glutathione (GSH): 5,5′-dithiobis(2-nitrobenzoic acid) (DTNB)-based quantification (412 nm); Superoxide dismutase (SOD): pyrogallol autoxidation method (420 nm). Splenic immune status was assessed via: Spleen coefficient: spleen-to-body weight ratio; Splenocyte proliferation: Alamar Blue assay on cultured splenocytes. Genotoxicity was measured by 8-hydroxy-2′-deoxyguanosine (8-OHdG) levels in DNA extracted from mouse brain (GenElute Kit, Sigma-Aldrich), quantified by enzyme-linked immunosorbent assay (ELISA) (New 8-OHdG Check, Japan Institute of Control of Aging).

#### Orthotopic intracranial tumor induction

GI-1 cells (1 × 10⁵ cells prepared as a stock suspension in 100 μL serum-free DMEM) were stereotactically implanted in mice (anesthetized with isoflurane) 35. A burr hole was drilled 2 mm right and 1 mm anterior to the bregma. A 3 μL suspension was injected into the right putamen at 1 μL/min using a Hamilton syringe. Isoflurane was maintained at 1.5-2% throughout the procedure. The skull was sealed with bone wax, and the skin sutured post-injection. Mice were monitored continuously post-surgery for signs of recovery.

#### Biodistribution and tumor targeting of HSLNs

One week post-implantation, tumor-bearing BALB/c-nu mice (n = 10/group) were divided into six groups: saline, curcin- indocyanine green (ICG) (curcin labeled with ICG using the ICG Labeling Kit - NH₂ (Dojindo, Japan) according to the manufacturer's protocol), and CdSe-loaded HSLNs (NT, TF, RGD, Dual). I.V. injections (0.5 mg/mouse) were given, and fluorescence/ICP-MS-based biodistribution was evaluated at 6, 24, and 48 h using the same protocol as described in the biodistribution methods section. Organ Cd content was normalized to tissue weight.

#### Therapeutic efficacy evaluation

Mice were randomly assigned to six groups: saline, free curcin (16 μg/mouse), and curcin-loaded HSLNs (NT, TF, RGD, Dual). I.V. administration was initiated 2 days post-tumor implantation (0.25 mg/mouse, equivalent to 16 μg curcin), repeated every other day for 36 days (18 injections). For ICR-nu mice, three groups were used: saline, NT, and Dual HSLNs.

Tumor assessment: Half the mice were euthanized mid-study for perfusion and brain extraction. Cryosections were analyzed by H&E and immunohistochemistry (β-galactosidase (β-gal), CD31, terminal deoxynucleotidyl transferase dUTP nick end labelling (TUNEL), Ki-67 (abcam)). A separate cohort was used for Annexin V (apoptosis) (Annexin Vivo 750, PerkinElmer), Integrisense (vasculature) (IntegriSense 750, PerkinElmer), and Evans Blue (BBB permeability) (Sigma-Aldrich) staining. Brains were imaged using Clairvivo OPT under an ICG filter or photographed (Evans Blue), and signals quantified from tumor versus normal regions. Image quantification was performed using ImageJ software (NIH, USA)*.* Survival analysis: Kaplan-Meier curves were plotted using GraphPad Prism v5.0. Mice were euthanized upon >20% body weight loss or signs of severe distress. Protein profiling: Tumor homogenates were analyzed with a Proteome Profiler Human Angiogenesis Array Kit (R&D Systems). Differential proteins were input into Search Tool for the Retrieval of Interacting Genes/Proteins (STRING) for interaction network analysis. Functional partners and co-expression modules were recorded.

#### Behavioral analysis

Behavioral assessments were conducted on BALB/c-nu and ICR-nu mice pre- and post-treatment, including general activity, feeding, and alertness (monitored every 48 h). Cylinder test 36: Mice were placed in a clear cylinder, and forelimb contacts during rearing were recorded for 5 minutes to evaluate motor asymmetry and sensorimotor function.

All behavioral scoring was performed blinded to treatment groups.

#### Neuroanatomical mapping

Brains were sectioned coronally and aligned to the Franklin and Paxinos mouse brain atlas 37. Tumor locations were identified and matched to functional domains (e.g., motor cortex, corpus callosum). Corresponding behavioral symptoms observed during survival analysis were cross-referenced with lesion sites.

### Statistical analysis

All quantitative data are presented as mean ± standard deviation (SD). Inter-group comparisons were performed using one-way or two-way analysis of variance (ANOVA), followed by Tukey or Bonferroni multiple-comparison tests as appropriate. Kaplan-Meier survival curves were analysed with the log-rank (Mantel-Cox) test**.** All statistics were calculated in GraphPad Prism v5.0 (GraphPad Software, USA), and differences were considered significant at *p* < 0.05. For in vivo efficacy and survival analyses, group sizes of n = 10 were used and considered appropriate for primary therapeutic endpoints. Proteomic (n = 3) and behavioral (n = 3-5) analyses were performed as exploratory mechanistic and functional correlates and were interpreted in conjunction with independent endpoints including tumor regression, histopathology, imaging, and survival outcomes.

## Results and Discussion

### Nanoparticle synthesis and characterization

**Figure 1:**
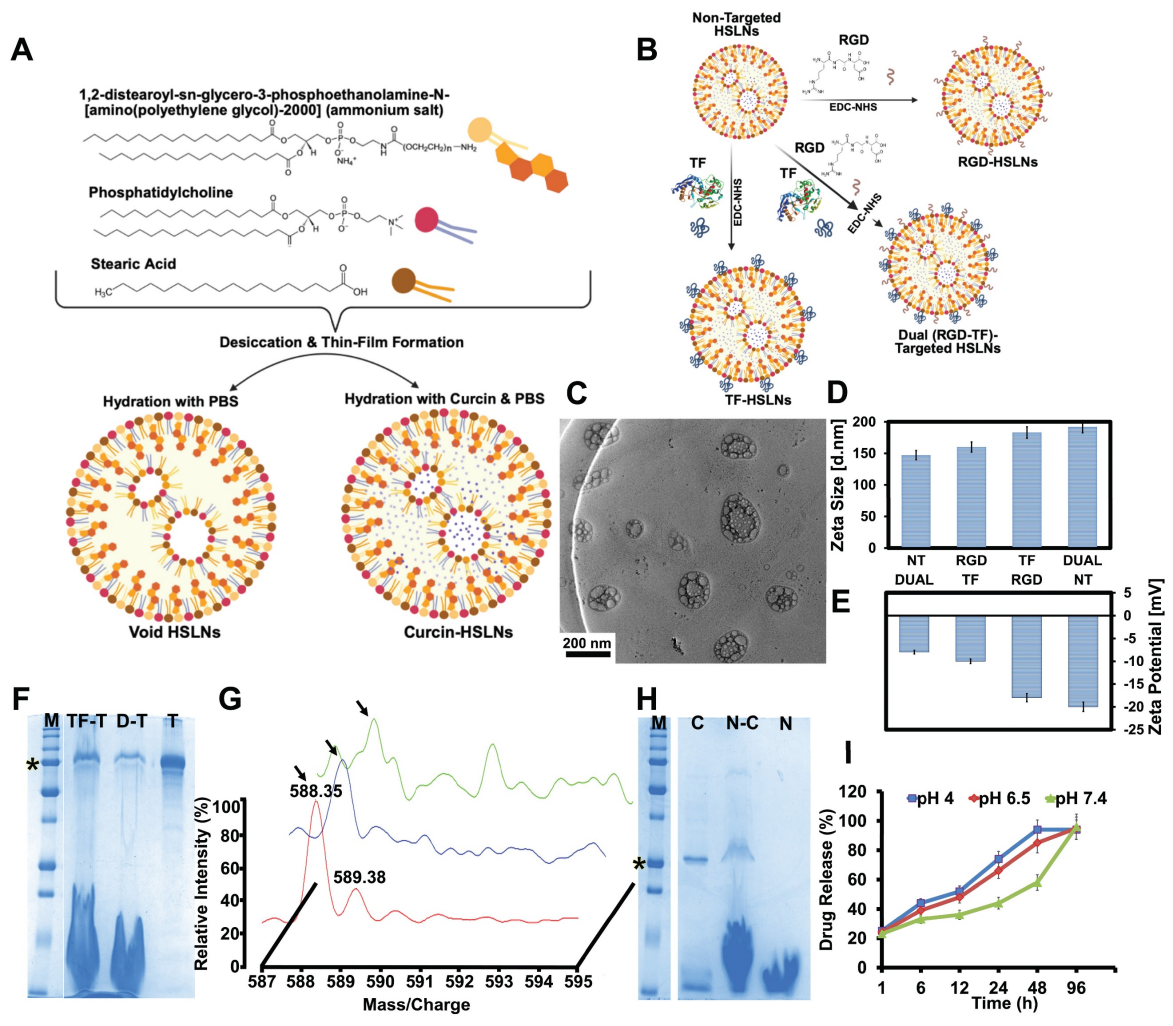
** Synthesis, physicochemical characterization, and pH-responsive release profile of curcin-loaded dual-targeted hybrid solid lipid nanoparticles (HSLNs). (A)** Schematic illustration of lipid components-DSPE-PEG(2000)-amine, phosphatidylcholine, and stearic acid- and the thin-film hydration method for generating void and curcin-loaded HSLNs. **(B)** Conjugation strategy for RGD peptide and TF to create RGD-HSLNs, TF-HSLNs, and Dual (RGD+TF) HSLNs via NHS-EDC chemistry. **(C)** TEM image showing spherical, monodisperse nanoparticles with vesicular substructures. Scale bar = 200 nm. **(D)** Hydrodynamic diameter of various HSLN formulations measured by DLS, ranging from 147-192 nm. **(E)** Zeta potential showing uniformly negative surface charge across all formulations (-20 to -8 mV), with ligand conjugation causing slight surface potential shifts. **(F)** SDS-PAGE confirming TF conjugation by detection of a ~79 kDa band in TF-HSLNs and Dual-HSLNs. (M: Marker, TF-T: TF-HSLNs, D-T: Dual-HSLNs, T: Transferrin, * represents 75 kDa M.W.) **(G)** MALDI-TOF mass spectrometry confirming RGD conjugation with a 588.35 m/z peak (arrow), indicative of successful peptide attachment. **(H)** SDS-PAGE validating curcin encapsulation, showing a 28 kDa band in curcin-loaded HSLNs. (M: Marker, C: Curcin, N-C: Curcin-HSLN, N: Void-HSLN, * represents 25 kDa M.W.). **(I)** *In vitro* release profile of curcin from HSLNs under acidic (pH 4.0, 6.5) and physiological (pH 7.4) conditions over 96 h. The biphasic profile includes an initial burst followed by sustained release; significantly higher release was observed under acidic conditions, simulating tumor microenvironments. Data in **(D-E, I)** are presented as mean ± SD (n = 3 independent replicates). Statistical analysis: one-way ANOVA with Tukey's post hoc test; *p* < 0.05 (*), *p* < 0.01 (**).

The HSLNs were fabricated through a modified thin-film hydration technique 26 **(Figure 1A-B)**. Morphological analysis using transmission electron microscopy (TEM) revealed a spherical to slightly ovoid structure with homogeneous dispersion and minimal aggregation **(Figure 1C; Figure S1A-H)**. Notably, the lipid matrix exhibited embedded vesicle-like substructures, which likely contribute to staged drug loading and controlled release behavior. These vesicular features increase the surface area for drug entrapment and provide internal aqueous compartments conducive to encapsulating hydrophilic biomacromolecules such as curcin.

The average hydrodynamic diameters, as measured by dynamic light scattering (DLS), ranged from 147 ± 18.6 nm (non-targeted; NT), 160 ± 19.8 nm (RGD-targeted; RGD), 183 ± 21.3 nm (TF-targeted; TF), to 192 ± 23.1 nm (RGD and TF-targeted; Dual) **(Figure 1D)**. These sizes are considered optimal for systemic circulation and for exploiting both the EPR effect and receptor-mediated transcytosis across the BBB, which typically favors particles in the 10-200 nm range 38. More recent work has emphasized that effective brain tumor delivery depends not only on particle size but also on rational surface engineering to promote receptor-mediated transport across the BBB, particularly in GBM and related aggressive gliomas, where vascular heterogeneity and active efflux mechanisms limit passive drug accumulation 39-41.

These considerations further highlight why conventional systemic therapies often fail to achieve durable intracranial exposure, reinforcing the need for multifunctional, BBB-aware nanocarrier strategies such as the one developed in this study.

Zeta potential analysis revealed a consistently negative surface charge across all formulations **(Figure 1E)**, with NT HSLNs exhibiting a value of around -20 mV, and TF, RGD, and Dual variants showing comparatively higher (less negative) values of -18 mV, -10 mV, and -8 mV, respectively. These values align with literature reports suggesting that a zeta potential in the range of -10 to -20 mV supports colloidal stability, limits protein adsorption, and reduces nonspecific uptake by the reticuloendothelial system (RES) 42, 43. The less negative surface potential observed for ligand-modified HSLNs may also facilitate better receptor interaction due to decreased electrostatic repulsion at the cellular interface.

Long-term colloidal stability was validated by storing the HSLNs at 4 °C in PBS for six months. Measurements post long-term incubation showed no significant changes in particle size or zeta potential, indicating strong physicochemical integrity. When incubated in 50% FBS, the nanoparticles maintained their hydrodynamic size and surface charge, demonstrating resistance to serum-induced aggregation and opsonization, attributes commonly associated with steric stabilization provided by DSPE-PEG-amine chains **(Table S1)** 44. After 24 h of serum exposure, HSLNs retained a stable hydrodynamic diameter (194 ± 30 nm) with no evidence of aggregation, accompanied by a modest increase in polydispersity (PDI < 0.5) and a mildly negative zeta potential (-10 mV). These results confirm preserved colloidal stability under physiologically relevant serum conditions. Successful decoration of the HSLN surface with TF and RGD was first confirmed qualitatively and then quantified. Qualitative confirmation relied on orthogonal read-outs: SDS-PAGE showed the diagnostic 79 kDa TF band exclusively in TF- and Dual-HSLNs **(Figure 1F)**, while MALDI-TOF MS of acid-cleaved nanoparticles revealed the expected RGD parent ion at m/z ≈ 588.35 in RGD- and Dual-HSLNs **(Figure 1G)**. For quantitative analysis, ligand densities were obtained independently from both the MALDI peak-area calibration and solution-phase mass balances. Peak integration against external standards indicated average surface coverages of 243 ± 18 TF or 125 ± 11 RGD molecules particle⁻¹ in the single-ligand formulations, and 121 ± 10 TF plus 68 ± 7 RGD molecules particle⁻¹ in the dual construct. These values were corroborated by micro-BCA assay of unbound TF and fluorescamine assay of residual peptide, which showed that in the dual-ligand formulations 72 ± 4% of the fed TF and 71 ± 6% of the fed RGD were covalently coupled; converting the bound masses to molecules per particle, using particle numbers derived from DLS size and dry-mass data, yielded densities within 8% of the MALDI estimates, confirming the accuracy of the conjugation and quantification workflow. Of the four dual-ligand ratios examined, the RGD₄:TF₆ composition produced the greatest GI-1 cell uptake and BBB transcytosis, implying that an intermediate RGD loading maximises integrin binding without sterically hindering transferrin-receptor engagement, in line with prior dual-targeting reports for CNS nanomedicines 45, 46.

Curcin, a type I RIP, was successfully encapsulated in the HSLNs, as confirmed by SDS-PAGE showing a distinct 28 kDa band **(Figure 1H)**. Drug loading and EE were calculated by lysing the nanoparticles and quantifying curcin content spectrophotometrically. The EE was 68 ± 9.8%, typical of protein-loaded lipid nanoparticles, which generally exhibit 30-60% efficiency 47, 48. Thus, the HSLN system demonstrates optimal loading potential for biologically active proteins, further supported by the amphiphilic matrix of stearic acid and lecithin and the vesicular substructure.

Drug release kinetics were interrogated under physiological (pH 7.4), tumor-relevant (pH 6.5), and highly acidic (pH 4.0) conditions to recapitulate systemic circulation, the extracellular TME, and endosomal compartments, respectively. Across all conditions, the HSLNs displayed a biphasic release behavior, comprising an early accelerated phase within the first 12 h, followed by a prolonged diffusion-dominated release regime extending to 96 h **(Figure 1I)**.

Quantitatively, acidic environments induced a pronounced kinetic acceleration of curcin release at early and intermediate stages. For instance, by 24 h, cumulative release reached approximately 74%, 66%, and 44% at pH 4.0, 6.5, and 7.4, respectively, with this separation further amplified at 48 h (~94%, ~85%, and ~58%, respectively). This behaviour is consistent with protonation-driven softening and partial destabilization of the hybrid lipid matrix, which lowers diffusional barriers and promotes more rapid payload liberation under acidic conditions.

Notably, despite these marked differences in release kinetics, cumulative release converged across all pH conditions by 96 h (~94-96%), indicating that environmental pH predominantly governs the rate of drug liberation rather than the total releasable fraction. This decoupling of kinetic control from ultimate release capacity represents a desirable design feature, enabling spatiotemporally accelerated drug availability within acidic tumor-associated or endosomal milieus, while maintaining controlled release under physiological conditions. Given that GBM, and by extension GSM, microenvironments typically exhibit extracellular acidification (pHₑ ≈ 6.1-6.8), such kinetically gated release behavior is expected to enhance intratumoral drug exposure without compromising systemic stability 49, 50.

**Figure 2:**
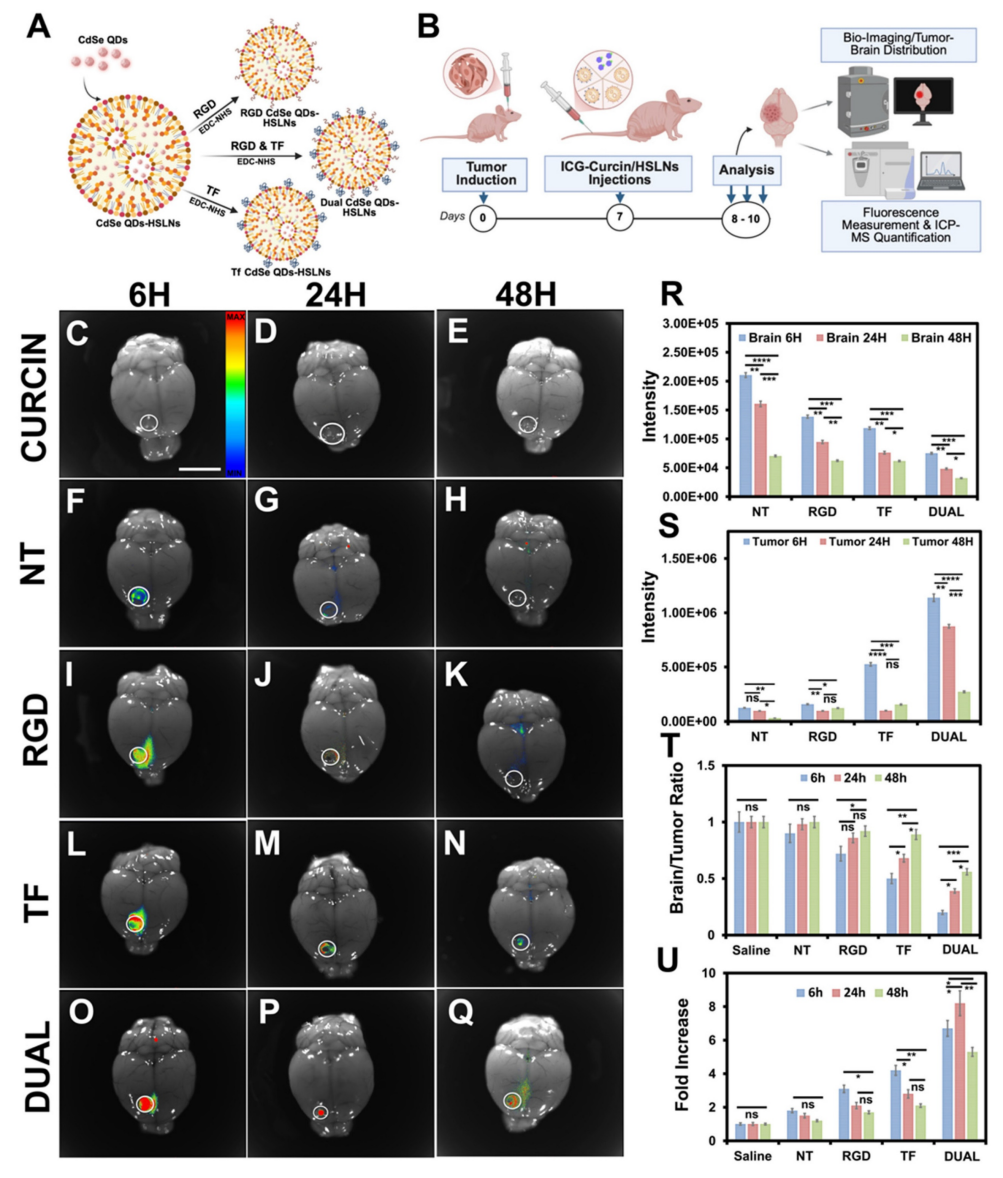
** Dual-targeted HSLNs demonstrate enhanced gliosarcoma tumor targeting, prolonged retention, and brain-tumor selectivity *in vivo*. (A)** Schematic representation of quantum dot (CdSe)-loaded HSLNs and respective ligand-functionalized formulations (NT, RGD, TF, Dual). **(B)** Experimental workflow for *in vivo* imaging and ICP-MS quantification in intracranial GI-1 gliosarcoma-bearing mice. ICG-curcin/HSLN formulations were administered via the tail vein; imaging and tissue analysis were performed at 6 h, 24 h, and 48 h post-injection. **(C-E)** Whole-brain fluorescence imaging of mice treated with free curcin (ICG-bound), showing negligible retention. (inset: fluorescent intensity map) **(F-H)** NT-HSLNs display weak, transient fluorescence. **(I-K)** RGD-HSLNs exhibit moderate initial tumor localization that diminishes by 48 h. **(L-N)** TF-HSLNs show a persistent signal through 48 h. **(O-Q)** Dual-HSLNs demonstrate the strongest and most sustained tumor-associated fluorescence signal. Tumor regions are circled in each image. Scale bar for **(C-Q)** = 5 mm **(R)** Quantification of fluorescence intensity in healthy brain tissue at 6, 24, and 48 h post-injection. **(S)** Fluorescence intensity at corresponding tumor sites. **(T)** Tumor-to-brain fluorescence ratio showing the highest selectivity for Dual-HSLNs. **(U)** Fold increase in tumor accumulation over time relative to saline. Data in **(R-U)** are mean ± SD (n = 10 mice/group). Statistical analysis: two-way ANOVA with Bonferroni post hoc test; ns = not significant, *p* < 0.05 (*), p < 0.01 (**), p < 0.001 (***), p < 0.0001 (****). Dual-HSLNs achieved ~7-fold higher tumor localization compared to NT-HSLNs and ~2-fold over single-ligand systems, reflecting superior cooperative receptor targeting and extended circulation. These findings establish Dual-HSLNs as highly tumor-specific and BBB-permeable CNS-targeted nanocarriers.

To facilitate real-time imaging and biodistribution studies, CdSe QDs were incorporated into the lipid core to create fluorescent HSLN-CdSe variants (**Figure 2A**). TEM analysis confirmed the preservation of nanoparticle morphology post-QD encapsulation **(Figure S2A-E)**, and photoluminescence spectra maintained emission maxima and bandwidth, indicating structural integrity and preserved quantum yield **(Figure S2F)**. XPS validated the presence of Cd and Se elements and ruled out any detectable cadmium leakage **(Figure S2G-I)**, addressing a major concern in QD-based diagnostics. Compared to conventional QD carriers like silica, polymeric micelles, or graphene oxide, which often require additional coatings to prevent cadmium ion leaching, the HSLNs demonstrated excellent retention and photostability, further enhancing their appeal as *in vivo* imaging agents 51.

Altogether, the synthesis and characterization data demonstrate that the dual-ligand HSLNs meet the critical physicochemical criteria for CNS-targeted nanotherapy. Their optimal size, high encapsulation efficiency, stability in serum-rich environments, targeted ligand architecture, pH-responsive drug release, and capacity for imaging payload delivery position them as highly promising theranostic candidates for GSM treatment.

### *In vitro* studies

Comprehensive *in vitro* assessments were conducted to evaluate the cytotoxicity, BBB permeability, and GSM-specific internalization of the curcin-loaded HSLNs. Alamar Blue viability assays showed that free curcin elicited strong cytotoxicity in a concentration-dependent manner across multiple cell types, including GSM (GI-1), human cortical neurons (HCN-1A), astrocytes (HA), brain endothelial cells (BEC), and pericytes (BPC) **(Figure S3)**. This broad cytotoxic effect reflects curcin's known mechanism of action as an RIP 25, 28, 29. By contrast, void HSLNs and HSLN-CdSe exhibited no significant cytotoxicity up to 500 µg/mL** (Figure S4A-B)**, supporting the inherent biocompatibility of the lipid-based carrier and demonstrating that encapsulated CdSe QDs pose negligible toxicity under physiological conditions.

Importantly, this finding distinguishes the HSLN-CdSe formulation from other QD-loaded carrier-based platforms, which often exhibit reactive oxygen species (ROS) generation, cadmium leakage, or photoluminescence quenching without extensive surface modifications 52, 53. Similarly, conventional fluorescent agents such as Cy5.5, DiR, fluorescein analogues, and ICG are prone to photodegradation, poor aqueous stability, and mitochondrial toxicity at high doses 54, 55. In contrast, HSLNs provide a robust lipidic encapsulation matrix that shields QDs from direct cellular interaction, preserves optical properties, and mitigates oxidative damage, thereby enabling safe use in theranostic applications targeting sensitive CNS tissues.

Curcin-loaded HSLNs (NT, RGD, TF, and Dual) were subsequently evaluated for GSM-selective cytotoxicity. GSM cells treated with Dual-targeted HSLNs displayed a significantly greater reduction in viability compared to single-ligand or NT formulations, while free curcin showed comparable cytotoxicity to NT and RGD formulations **(Figure S4C)**. The enhanced cytotoxicity observed for Dual HSLNs is consistent with the overexpression of both transferrin receptors (TfR) and integrins (αvβ3/α5β1) in GSM cells, which enables synergistic multivalent binding and endocytosis 56, 57.

To mechanistically validate internalization, flow cytometry was performed on GI-1 cells incubated with fluorescently labelled curcin-FITC and HSLN-CdSe variants. Dual HSLNs consistently exhibited the highest cellular uptake, with 4:6 RGD:TF ligand ratios showing optimal internalization efficiency **(Figure S4D-F)**. This stoichiometric balance likely prevents receptor saturation while promoting multivalent interactions with both TfR and integrin targets. Previous work on dual-ligand nanoplatforms has demonstrated that fine-tuning ligand densities significantly improves binding avidity, receptor clustering, and cellular uptake 58. Thus, the optimized Dual HSLNs here not only validate this principle but also align with prior synthesis data supporting the 4:6 ratio as optimal for GSM targeting.

To evaluate BBB penetration, an *in vitro* model was established using co-cultures of BEC and BPC on transwell membranes **(Figure S5A)**. TEER values exceeded 300 Ω·cm², confirming the formation of tight junctions representative of the physiological BBB. FITC-dextran and inulin permeability assays further validated barrier integrity **(Figure S5A-C)**. Upon exposure to curcin-FITC and HSLN-CdSe formulations, Dual HSLNs demonstrated the highest translocation efficiency, particularly those with 4:6 RGD:TF ratios **(Figure S5D-E)**. This enhancement is attributable to the cooperative engagement of TfR- and integrin-mediated transport mechanisms, offering orthogonal transcytosis routes 56.

Therapeutic relevance was assessed using a secondary *in vitro* BBB model with GI-1 cells seeded in the basolateral chamber **(Figure S5F)**. After 4 hours of nanoparticle exposure, inserts were removed, and cell viability in the basolateral compartment was evaluated after 72 hours. Dual-targeted HSLNs elicited the greatest reduction in post-BBB tumor cell viability relative to all other groups **(Figure S5G)**, indicating both effective BBB penetration and biologically active curcin release at the tumor interface. Importantly, this post-transcytosis cytotoxicity underscores the functional delivery of encapsulated protein and is a key performance benchmark for CNS-targeted nanoparticle systems. Also, TEER values post-treatment with curcin-loaded HSLNs did not show a significant decline (353 Ω·cm²) compared to baseline, indicating that the nanoformulations preserved barrier integrity. In contrast, free curcin treatment led to a noticeable reduction in TEER (224 Ω·cm²), likely due to its non-specific cytotoxicity toward BEC and BPC. These results underscore the protective advantage of HSLN encapsulation in maintaining BBB model structure while ensuring tumor-directed cytotoxicity.

To mechanistically contextualize the superior *in vitro* performance of the dual-functionalized HSLNs observed across cellular uptake, BBB transcytosis, and post-BBB cytotoxicity assays **(Figure S5)**, receptor expression profiling and targeted inhibition studies were conducted. Flow-cytometric analysis confirmed that GI-1 and U-87MG cells co-express transferrin receptor (TfR) together with integrin α5 and β3 **(Figure S6A-B)**, whereas non-tumor brain cells predominantly expressed TfR with comparatively lower integrin levels **(Figure S6C-D)**, providing a molecular basis for the dual-ligand targeting strategy. Functional receptor-blocking experiments using dual-functionalized HSLNs revealed that inhibition of either TfR or integrins alone **(Figure S7B-C, G-H)** did not substantially diminish nanoparticle uptake relative to the no-blocking condition **(Figure S7E-J)**, whereas simultaneous blockade of both receptor classes resulted in a marked suppression of intracellular fluorescence **(Figure S7D-I)**. This indicates that TfR- and integrin-mediated interactions act cooperatively and in a partially compensatory manner, enabling sustained cellular internalization even when one pathway is restricted. Importantly, this cooperative uptake behavior is fully consistent with earlier quantitative uptake and BBB transcytosis data, where dual-functionalized HSLNs consistently outperformed mono-ligand formulations, supporting the concept that dual targeting enhances robustness against receptor heterogeneity rather than reliance on a single dominant entry route. Complementary endocytic pathway inhibition studies **(Figure S8)** further demonstrated that nanoparticle internalization was strongly attenuated under energy-restricted conditions **(Figure S8E-J)** and upon clathrin-mediated endocytosis inhibition **(Figure S8C-H)**, while macropinocytosis inhibition **(Figure S8D-I)** had minimal effect, identifying a dominant TfR-associated clathrin pathway that supports productive intracellular trafficking and enhanced BBB transcytosis. Collectively, these mechanistic findings provide a coherent explanation for the enhanced intracellular delivery, post-BBB cytotoxicity, and superior therapeutic efficacy of dual-functionalized HSLNs observed throughout the *in vitro* evaluation. Altogether, the *in vitro* studies confirm the dual HSLNs' multifunctionality-combining efficient GSM targeting, BBB penetration, and curcin-mediated cytotoxicity. The superior performance of 4:6 RGD:TF HSLNs validates the importance of ligand stoichiometry and receptor synergy in CNS nanotherapy design. These data establish a strong rationale for advancing the Dual HSLNs into *in vivo* evaluation for GSM treatment.

### Biocompatibility and systemic safety

Following validation of the *in vitro* efficacy and BBB permeability, systemic biocompatibility and biodistribution of the HSLNs were evaluated in healthy BALB/c-nu mice. Void HSLNs administered intravenously on alternate days for 20 days (10 doses total, n = 10) did not induce any noticeable physiological abnormalities, behavioral changes, or mortality (**Scheme S1**). Body weight of treated mice remained comparable to saline-treated controls, supporting the systemic tolerance of the nanoformulation **(Figure S9A)**.

Histological examination of major organs (brain, heart, liver, kidney, spleen, and lungs) from HSLN-treated and control groups revealed no signs of inflammation, necrosis, or tissue degeneration **(Figure S9B-M)**. These findings were supported by unaltered serum biochemical profiles **(Figure S9N-O)**, indicating normal hepatic, renal, and hematological function. The absence of hepatosplenic toxicity was especially notable, considering the RES's preferential uptake of intravenously administered nanoparticles 59.

Antioxidant profiling of liver homogenates further confirmed the safety of HSLNs. Levels of oxidative stress markers such as LPO and antioxidant defenses including GSH, SOD, GPX, and GR remained within physiological limits **(Figure S9P)**. Notably, 8-OHdG, a key biomarker of oxidative DNA damage, was undetectable in the brains of HSLN-treated mice. These outcomes underscore the ability of the HSLNs to avoid oxidative tissue damage, a significant advantage over several other nanoparticles that often trigger ROS accumulation and mitochondrial dysfunction 60, 61.

Spleen coefficient and splenocyte proliferation assays, key indicators of immunotoxicity and lymphoid activation, showed no significant differences between void HSLN-treated and untreated groups. In contrast, mice treated with free curcin exhibited a marked increase in spleen coefficient, consistent with its known immunostimulatory effects as an RIP. These findings reinforce the premise that encapsulation not only improves the delivery and pharmacokinetics of curcin but also attenuates its systemic off-target effects.

To assess biodistribution, curcin-free NT HSLN-CdSe were injected into healthy mice and tracked using fluorescence imaging and quantified using ICP-MS. Whole-body imaging 6 h post-administration revealed intense systemic fluorescence **(Figure S9Q)**, confirming the wide distribution of the NT HSLNs. ICP-MS data indicated that approximately 28% of the injected dose remained in circulation (blood), while organ-specific accumulation was highest in the liver (~8%), followed by spleen and kidneys **(Figure S9R)**. These data are consistent with classical RES-mediated nanoparticle clearance 59, 62.

Importantly, no signal was detected in the brain tissue of healthy mice, supporting the selectivity of the nanoparticle for pathological tissue environments. These results compare favorably with previous reports on polymeric or dendrimer-based systems, which frequently show significant off-target deposition in the liver and spleen, contributing to dose-limiting toxicity 62, 63.

Overall, the biocompatibility results affirm that the HSLN platform, whether void, curcin-loaded (targeted versions), or imaging-enabled, has an excellent safety profile, enabling its further development for GSM therapy. These outcomes, when combined with their therapeutic performance and targeting specificity, suggest strong translational potential for both clinical and diagnostic CNS applications.

### Biodistribution and tumor targeting

The *in vivo* biodistribution and tumor-targeting efficiency of curcin-free HSLNs (HSLN-CdSe) were assessed in intracranial GSM-bearing mice using whole-body fluorescence imaging **(Figure 2B; Figure S10A-O)**. Six hours post-injection, strong fluorescence signals were observed across the head and thoracoabdominal regions **(Figure S10D,G,J,M)**, reflecting rapid systemic distribution and circulatory persistence. Fluorescence intensity in peripheral tissues declined progressively over 24-48 h **(Figure S10F,I,L,O)**, consistent with RES-mediated clearance and nanoparticle elimination kinetics.

*Ex vivo* imaging of mice treated with free curcin (ICG-bound) showed predominant hepatic and renal fluorescence, suggesting rapid metabolism and non-specific biodistribution **(Figure S10A′-B′; Figure S11A-C, Figure 2C-E)**. Conversely, all HSLN formulations, NT, RGD, TF, and Dual, exhibited detectable accumulation in the brain tumor region, confirming successful BBB traversal and selective tumor tropism **(Figure 2F-Q)**. This ability to localize within the GSM niche, despite heterogeneous BBB permeability, marks a major advancement for CNS-directed nanotherapies 56, 64.

Among the variants, Dual HSLNs demonstrated the most robust and persistent fluorescence in the tumor area at 48 h post-injection **(Figure 2Q)**, whereas single-ligand HSLNs (RGD and TF) showed moderate but shorter-lived retention **(Figure 2K,N)**. These results indicate that the dual-targeting configuration substantially improves intratumoral residency, a critical factor for maximizing therapeutic payload delivery.

ICP-MS analysis of Cd content in tumor and normal brain tissues revealed that Dual HSLNs demonstrated minimal accumulation in healthy brain parenchyma **(Figure 2R)** and maximal localization within tumor sites **(Figure 2S)**, resulting in the highest tumor-to-brain (T/B) accumulation ratio among all treatment groups **(Figure 2T)**. This pronounced spatial selectivity highlights the formulation's tumor-targeting capability and favorable safety profile.

Off-target distribution patterns were consistent with known RES clearance profiles. NT HSLNs primarily accumulated in the liver, spleen, and lungs, while RGD HSLNs achieved modest tumor localization. TF HSLNs showed balanced distribution across tumor and systemic compartments. Strikingly, Dual HSLNs achieved a ~7-fold increase in tumor accumulation compared to NT and a 2-fold increase relative to single-ligand systems **(Figure 2U)**, highlighting the additive benefit of integrin and transferrin dual-targeting.

Compared to reported CNS-targeted nanocarriers, such as angiopep-conjugated polymers, lactoferrin-liposomes, or cell-penetrating peptide nanoparticles, the present Dual HSLNs demonstrated superior tumor-specific localization and extended systemic circulation, likely attributed to optimized ligand stoichiometry and PEGylation-driven steric stabilization 65, 66. Previous studies using angiopep-2 PEG-PLGA nanoparticles reported 2-3-fold increases in brain uptake relative to unmodified NPs, but often lacked tumor specificity or exhibited lower retention time in orthotopic GBM models 67. Similarly, transferrin-liposome hybrids and lactoferrin-modified micelles achieved moderate T/B ratios (~1.5-2.5), but with faster systemic clearance and limited penetration depth 68, 69. In contrast, Dual HSLNs displayed a significantly higher T/B ratio and superior persistence at the tumor site, sustaining localization beyond 48 h.

Recent dual-targeted nanoformulations for GBM, such as RGD-Angiopep-modified lipid NPs and IL-13Rα2/EGFR bispecific dendrimers, have shown improved intratumoral accumulation but were often hampered by suboptimal ligand ratios, competitive binding, or unfavorable pharmacokinetics 70, 71. The Dual HSLNs in this study address these limitations through ratio-optimized conjugation, enabling cooperative engagement of both integrin and transferrin receptors without saturation or steric hindrance. This balance not only facilitates effective BBB translocation but also enhances endocytic uptake within heterogeneous tumor zones, outperforming most monofunctional and even some multifunctional nanocarriers developed for GBM or GSM.

Notably, the extended circulation time (6-fold higher than NT) observed with Dual HSLNs **(Figure S11K)** enhances both EPR-driven accumulation and active transcytosis. This dual-mode delivery ensures sufficient temporal and spatial exposure of the therapeutic cargo to tumor cells while minimizing systemic toxicity.

Overall, the biodistribution studies affirm that Dual HSLNs possess an ideal pharmacokinetic profile for CNS tumors, characterized by high tumor retention, minimal off-target accumulation, and prolonged systemic availability. This formulation clearly outperforms conventional single-target systems and represents a meaningful advancement in site-specific GSM drug delivery.

### Therapeutic efficacy in orthotopic gliosarcoma models

**Figure 3:**
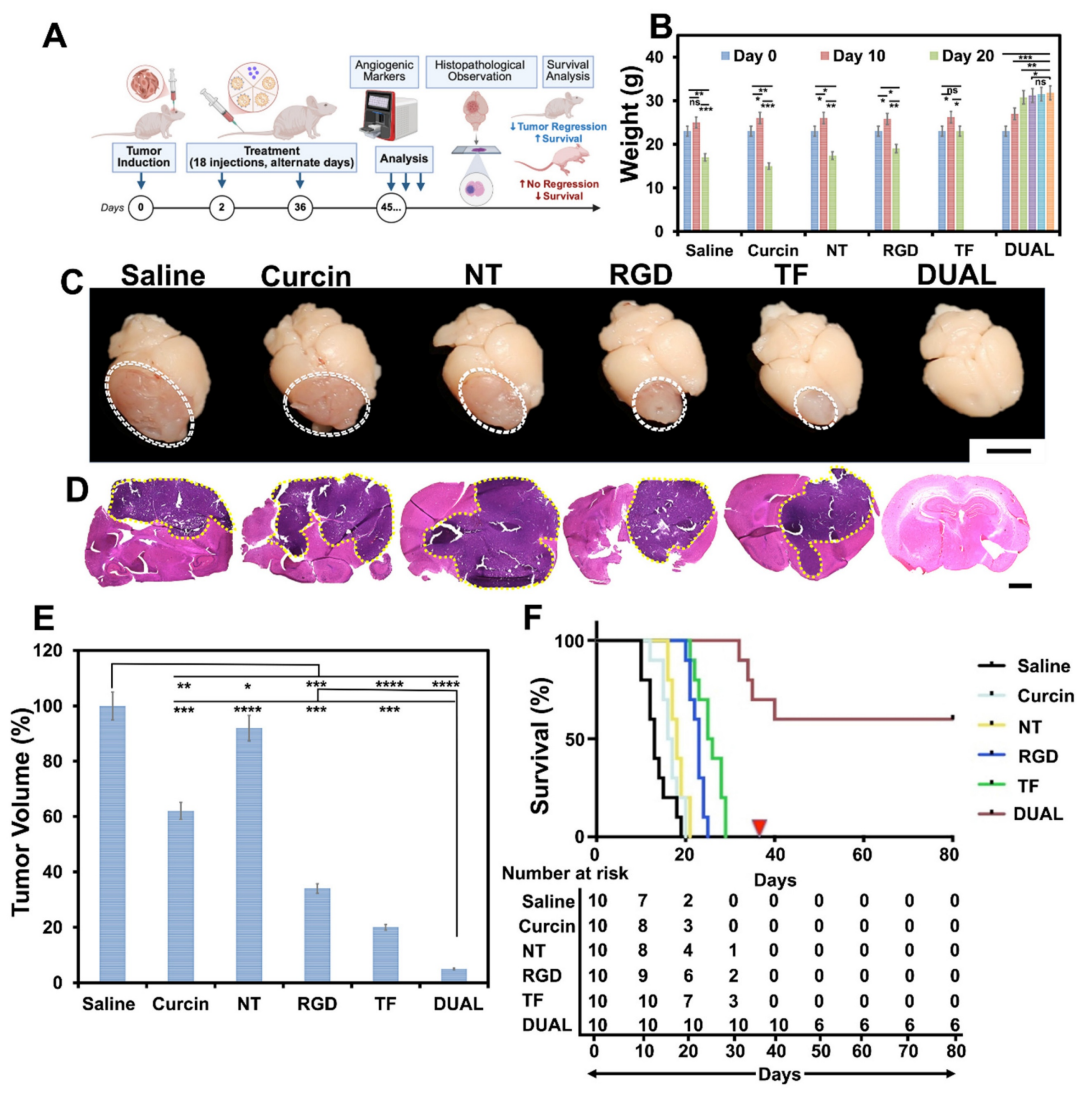
** Dual-targeted curcin-loaded HSLNs induce profound tumor regression and survival extension in orthotopic gliosarcoma models. (A)** Schematic of the treatment regimen. BALB/c-nu mice bearing intracranial GI-1 gliosarcomas were treated intravenously with saline, free curcin, or curcin-loaded HSLNs (NT, RGD, TF, Dual) every other day (18 injections total, 0.6 mg/kg curcin per dose). Survival and tumor progression were evaluated up to day 45. **(B)** Longitudinal body weight measurements at days 0, 10, and 20 indicate health status during treatment. Dual-HSLN-treated mice gained weight, while others experienced progressive decline. **(C)** Representative macroscopic brain images showing tumor bulk in control and single-ligand groups (white-dashed outlines), with near-complete clearance in the Dual group. Scale bar = 5 mm.** (D)** H&E-stained brain sections showing extensive tumor invasion (purple) in all groups except Dual, which exhibited minimal or no residual tumor (yellow-dotted outlines). Scale bar = 1 mm. **(E)** Quantitative tumor volume analysis by histomorphometry. Dual-HSLNs achieved ~95% reduction, significantly surpassing TF (~80%) and RGD (~71%) groups. **(F)** Kaplan-Meier survival analysis. Median survival: Saline = 14 d; Curcin = 17 d; NT = 19 d; RGD = 22 d; TF = 25 d; Dual = 38 d. The red triangle indicates the endpoint. Data in **(B, E)** are presented as mean ± SD (n = 10 mice per group). Statistical analysis: one-way ANOVA with Tukey's post hoc test for **(B, E)**; log-rank (Mantel-Cox) test for **(F)**; ns = not significant, *p* < 0.05 (*), p < 0.01 (**), p < 0.001 (***), p < 0.0001 (****). These results confirm that Dual-HSLNs significantly outperform single-ligand or untargeted systems in inducing tumor regression and extending survival, due to cooperative receptor targeting, improved BBB penetration, and reduced systemic toxicity.

The therapeutic performance of curcin-loaded HSLNs was evaluated using an orthotopic GSM mouse model (BALB/c-nu) **(Figure 3A)**. Animals were treated intravenously every other day for 36 days (18 doses) with saline, free curcin, or curcin-loaded HSLNs (NT, RGD, TF, or Dual), each administered at an equivalent curcin dose of 0.6 mg/kg. The selected curcin dose was based on tolerability considerations. Curcin, as a type I RIP, exhibits dose-limiting systemic toxicity when administered in free form at higher concentrations. A conservative dose was therefore chosen to permit repeated intravenous administration while minimizing systemic adverse effects, enabling evaluation of whether nanoparticle encapsulation and targeting could enhance therapeutic efficacy without increasing toxicity. Mice treated with Dual HSLNs exhibited steady weight gain and no observable signs of distress, whereas animals in all other treatment groups showed progressive weight loss and declining health, consistent with ongoing tumor progression **(Figure 3B)**. Macroscopic inspection of excised brains revealed extensive reddish tumor masses in the saline, free curcin, NT, RGD, and TF groups. By contrast, brains from the Dual HSLN cohort showed minimal or absent visible tumor **(Figure 3C)**. Histological analyses supported these findings, tumors in control and single-ligand groups infiltrated large brain regions, whereas 60% of Dual HSLN-treated mice showed complete tumor regression **(Figure 3D)**, with only minor remnants in the remaining 40%. Quantitative image analysis demonstrated ~95% reduction in tumor volume in the Dual group **(Figure 3E)**, outperforming TF (~80%) and RGD (~71%) HSLNs.

Kaplan-Meier survival analysis highlighted the significant survival advantage conferred by Dual HSLNs: median survivals of 14 (saline), 17 (free curcin), 19 (NT), 22 (RGD), and 25 (TF) days were recorded, while the Dual group showed a median survival of 38 days, with 60% of mice surviving beyond the experimental timeline without signs of relapse **(Figure 3F).**

These outcomes corroborate prior biodistribution data and reflect the cumulative benefits of enhanced BBB penetration, increased tumor retention, and receptor-specific uptake. Compared to previously reported single-ligand nanoparticle therapies or chemotherapeutic-loaded micelles that achieve partial tumor suppression (typically 50-70%) and survival extensions of 20-30 days 72, 73, the Dual HSLNs demonstrate a more durable and profound anti-tumor response. While certain multi-receptor-targeted nanosystems have achieved tumor regression and moderate survival gains 74, 75, few have matched the combined remission and extended survival observed here.

**Figure 4:**
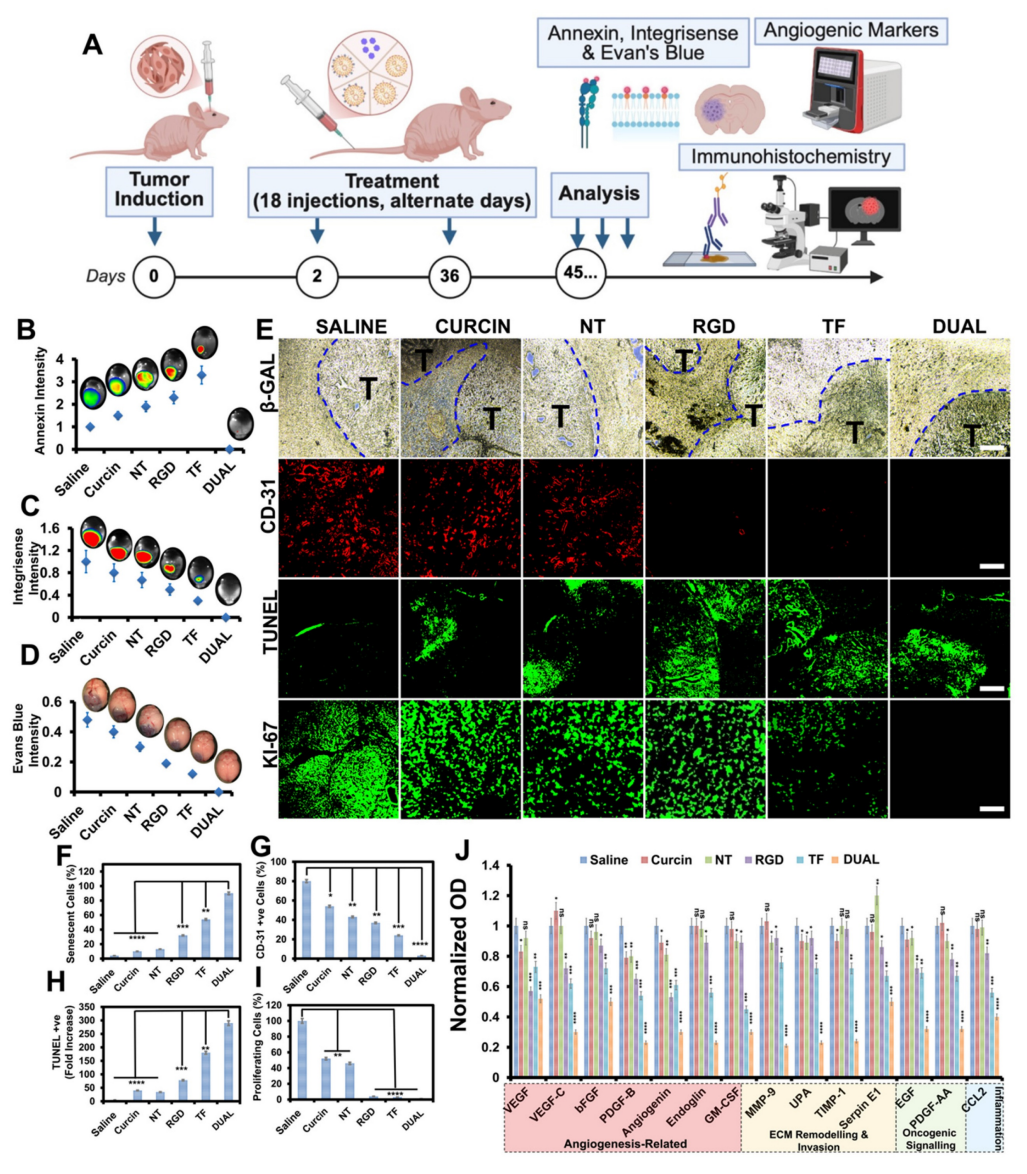
** Dual-targeted HSLNs induce apoptosis, inhibit angiogenesis and proliferation, and modulate key tumorigenic pathways in gliosarcoma-bearing mice. (A)** Schematic representation of the experimental workflow for mechanistic assessment, including tumor induction, 36-day treatment, and multi-modal analysis of apoptosis, vascular remodeling, and protein expression. **(B-D)** Quantification of whole-brain imaging intensities post-injection of molecular probes: **(B)** Annexin V (apoptosis), **(C)** Integrisense-750 (angiogenesis), and **(D)** Evans Blue (BBB permeability). Dual-HSLN treatment elicited the highest apoptosis, the lowest angiogenic activity, and restored BBB integrity. **(E)** Immunohistochemical and fluorescent staining of brain tumor sections. Top to bottom: β-galactosidase (senescence), CD31 (microvessel density), TUNEL (apoptotic index), and Ki-67 (proliferation). Tumor regions marked as 'T'. Dual-HSLNs exhibited high senescence, minimal angiogenesis, strong apoptosis, and near-complete suppression of proliferation. Scale bar = 100 µm **(F-I)** Quantitative analysis of marker expression: **(F)** β-gal staining, **(G)** percentage of CD31-positive cells, **(H)** percentage of TUNEL-positive cells, and **(I)** percentage of Ki-67-positive cells. **(J)** Proteomic profiling showing normalized expression intensities of angiogenic/tumorigenic protein markers across treatment groups. Dual-HSLNs downregulated VEGFA, MMP9, EGF, PDGFB, SERPINE1, and others more profoundly than single-ligand HSLNs. Data in **(B-D, F-J)** are presented as mean ± SD (n = 3-5 mice/group). Statistical analysis: one-way ANOVA with Tukey's post hoc test; ns = not significant, *p* < 0.05 (*), p < 0.01 (**), p < 0.001 (***), p < 0.0001 (****). These results confirm that Dual-HSLNs reprogram the tumor microenvironment by enhancing apoptosis, suppressing neovascularization, blocking cell proliferation, and collapsing cooperative oncogenic networks, contributing to durable tumor suppression observed *in vivo*.

Mechanistically, the antitumor efficacy was supported by apoptotic and angiogenic analyses **(Figure 4A)**. Annexin imaging showed minimal residual apoptotic signal in the Dual group **(Figure 4B)**, consistent with extensive apoptosis/necrosis followed by near-complete tumor clearance at the time of analysis, while integrisense vascular imaging showed that untreated and single-target groups retained dense vasculature networks, in contrast to the Dual group, where vascular signal was nearly abolished **(Figure 4C)**. Evans blue dye extravasation studies further demonstrated that BBB integrity was preserved in Dual-treated animals but compromised in all others **(Figure 4D; Figure S12)**, indicating localized, tumor-specific vascular remodeling without peripheral neurotoxicity.

Immunohistochemical staining for β-galactosidase (senescence), CD31 (angiogenesis), TUNEL (apoptosis), and Ki-67 (proliferation) reinforced this therapeutic profile: Dual HSLN-treated brains displayed high senescence, reduced microvascular density, enhanced apoptotic index, and an absence of proliferative markers **(Figure 4E-I)**, suggesting functional tumor dormancy and shutdown of proliferative signaling 76.

Molecular profiling via proteomics analysis **(Figure 4J)** provided a deeper mechanistic understanding of the treatment efficacy. **Table S2** lists 14 key angiogenic and tumorigenic proteins that were significantly downregulated in the Dual HSLN group compared to all other cohorts. These include VEGFA, VEGFC, FGF2, ANG, PDGFB, MMP-9, PLAU, CSF2, TIMP1, EGF, SERPINE1, and others. The reduction of these markers correlates with the anti-angiogenic, anti-invasive, and anti-proliferative histological findings 77.

**Table S3** presents the protein-protein interaction (PPI) network derived from STRING analysis, which reveals a densely connected cluster of co-regulated targets. Central hubs such as VEGFA, PDGFB, MMP-9, and SERPINE1 were identified as key mediators of GSM progression, involved in vascular proliferation, extracellular matrix degradation, and tumor invasiveness. Proteins like TIMP1 and PLAU, linked to tissue remodeling and migration, were shown to interact closely with growth factors such as FGF2 and EGF, amplifying oncogenic feedback loops under untreated conditions.

These molecular hubs were selectively dismantled following Dual HSLN treatment, suggesting that the platform induces collapse of cooperative oncogenic signaling rather than simply suppressing individual pathways. This network-wide inhibition aligns with the phenotypic observations of tumor regression, vascular pruning, and survival extension 78, 79.

Furthermore, **Table S4** details phylogenetic and expression co-occurrence analyses, highlighting the evolutionary conservation and functional interdependence of these suppressed proteins across glioma models. Such convergence reinforces the systems-level impact of the Dual HSLNs, offering mechanistic substantiation for their unique efficacy in GSM therapy 80, 81.

In sum, these findings illustrate the profound molecular rewiring induced by Dual HSLNs, translating into not just tumor size reduction, but lasting microenvironmental normalization and durable therapeutic benefit.

### Validation of dual HSLNs in the ICR-nu model

**Figure 5:**
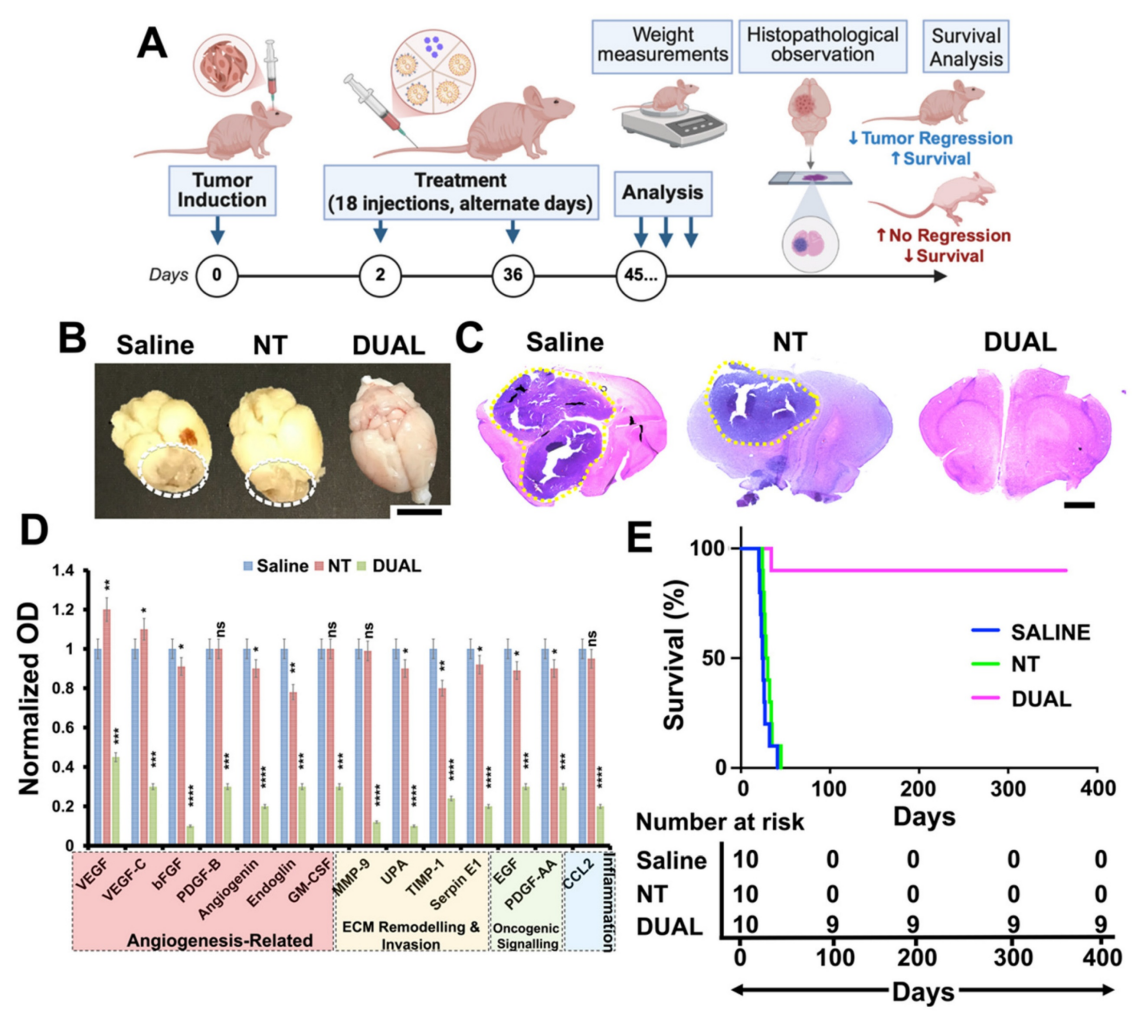
** Dual-HSLNs demonstrate reproducible tumor clearance and long-term survival in an independent ICR-nu gliosarcoma model. (A)** Experimental timeline for validation in ICR-nu mice bearing intracranial GI-1 tumors. Mice received 18 intravenous injections (0.6 mg/kg curcin) over 36 days, followed by assessment of tumor burden, angiogenic markers, and survival. **(B)** Macroscopic brain images from saline-, NT-HSLN-, and Dual-HSLN-treated mice. Tumor bulk is apparent in the saline and NT groups (dashed outlines), but absent in the Dual group. Scale bar = 5 mm. **(C)** H&E-stained brain sections show extensive tumor infiltration in saline and NT mice (yellow-dashed areas), whereas Dual-treated brains appear tumor-free. Scale bar = 1 mm. **(D)** Quantitative proteomic profiling of angiogenic and tumorigenic proteins. Dual-HSLNs significantly downregulated VEGFA, MMP9, PDGFB, GM-CSF, and related markers, consistent with prior BALB/c-nu results. **(E)** Kaplan-Meier survival analysis: all saline and NT mice died within 45 days, whereas 90% of Dual-treated mice survived past 100 days without relapse. Data in **(D)** are mean ± SD (n = 3-5 mice per group). Statistical analysis: one-way ANOVA with Tukey's post hoc test (d); log-rank (Mantel-Cox) test (e); ns = not significant, *p* < 0.05 (*), p < 0.01 (**), p < 0.001 (***), p < 0.0001 (****). These results confirm the systemic efficacy, translational reproducibility, and durable anti-tumor benefit of Dual-HSLNs in a genetically distinct gliosarcoma model.

To confirm the reproducibility and strain-independent efficacy of Dual HSLNs, therapeutic evaluations were extended to an independent cohort of ICR-nu mice bearing intracranial GSM tumors **(Figure 5A)**. Unlike BALB/c-nu mice, which are inbred and genetically uniform, the ICR-nu model is derived from an outbred background and exhibits greater genetic heterogeneity and physiologic variability. This heterogeneity better mimics the genetic diversity encountered in human patient populations, making this validation step essential for demonstrating the translational robustness and broad-spectrum efficacy of Dual HSLNs across genetically distinct murine hosts.

Following the previously established protocol (18 intravenous administrations of curcin-loaded Dual HSLNs at 0.6 mg/kg), the ICR-nu model yielded consistent outcomes. Nearly all mice in the saline and NT HSLN groups succumbed to tumor progression during the study period. In contrast, only one mortality occurred in the Dual HSLN group. Gross brain inspections revealed large tumor burdens in saline and NT groups, while brains from the Dual-treated cohort lacked discernible lesions **(Figure 5B)**. These macroscopic findings were supported by H&E staining, which confirmed the absence of tumor foci in the Dual group **(Figure 5C)**, suggesting near-complete therapeutic resolution.

Protein expression profiling of ICR-nu brain tissues mirrored the downregulation trends observed in BALB/c-nu mice, with a marked reduction in angiogenic and oncogenic markers in the Dual HSLN group **(Figure 5D)**. This molecular consistency across models highlights the systemic mechanism of tumor suppression induced by the formulation. It should be mentioned here that, although curcin selection was not guided by GSM-specific molecular profiling performed a priori, the proteomic alterations observed here provide strong *post hoc* validation for its suitability in GSM therapy. The coordinated downregulation of angiogenic, invasive, and extracellular matrix-associated pathways **(Figure 4J, Figure 5D; Tables S2-S4)** aligns with known molecular drivers of the mesenchymal and therapy-resistant phenotype characteristic of GSM. These findings suggest that curcin, delivered via an optimized brain-targeted nanocarrier, exerts broad systems-level suppression of tumor-supportive networks rather than targeting a single oncogenic pathway, which is particularly relevant for heterogeneous and chemoresistant GSMs.

By day 45 post-implantation, survival outcomes further validated efficacy: all saline and NT-treated mice had expired, whereas 90% of Dual HSLN-treated animals remained alive without signs of relapse or neurological deficit **(Figure 5E)**. Median survival was not reached for Dual-HSLN-treated ICR-nu mice during the study period, with ~90% survival maintained beyond 100 days. This result represents a substantial improvement over the 60% survival rate observed in the BALB/c-nu cohort, indicating enhanced or equivalent therapeutic benefit in a more genetically diverse host.

While comparative outcomes from BALB/c-nu mice already established the performance of Dual HSLNs against monoligand nanocarriers and free curcin, the present validation adds further support by demonstrating that the survival extension and tumor eradication observed are not model-specific phenomena. Although dual-ligand nanoparticles have improved tumor penetration in glioma/GBM models, virtually none of the available studies, and none in bona-fide GSM models, report sustained remission or survival beyond 60 days (in this case ~400 days) 70, 73, 74, 82.

Emerging gene-editing therapies, including nanoparticle-mediated delivery of clustered regularly interspaced short palindromic repeats-CRISPR-associated protein 9 (CRISPR-Cas9) or small interfering RNAs (siRNAs), offer additional avenues for glioma management. However, issues related to immunogenicity, off-target effects, and limited intracerebral penetration continue to hinder clinical progression 83, 84.

In contrast, the current platform leverages a dual-receptor mechanism and an enzymatically active protein payload (curcin) to induce multi-level suppression of GSM pathology with minimal systemic toxicity.

Collectively, the results from both BALB/c-nu and ICR-nu models underscore the translational promise of Dual HSLNs in GSM therapy. Their ability to reproducibly eliminate tumors, normalize the tumor microenvironment, and prolong survival in distinct animal models supports their continued preclinical advancement.

### Behavioral and neurological outcomes

Neurological and behavioral impairments represent some of the most clinically burdensome effects of GSM (and GBM) progression and treatment. Tumor development in the brain parenchyma can compromise sensorimotor circuits, cognitive capacity, and emotional regulation. Furthermore, conventional treatments, including radiotherapy, chemotherapy, and even some nanomedicines, are known to exacerbate neurotoxicity or inadequately address functional recovery. Despite this, most preclinical GSM studies continue to emphasize tumor regression and survival metrics, often neglecting behavioral and neurological outcomes 85, 86. To address this gap, the current study incorporated a dedicated behavioral and neuroanatomical analysis to evaluate the broader therapeutic relevance of Dual HSLN therapy.

**Figure 6:**
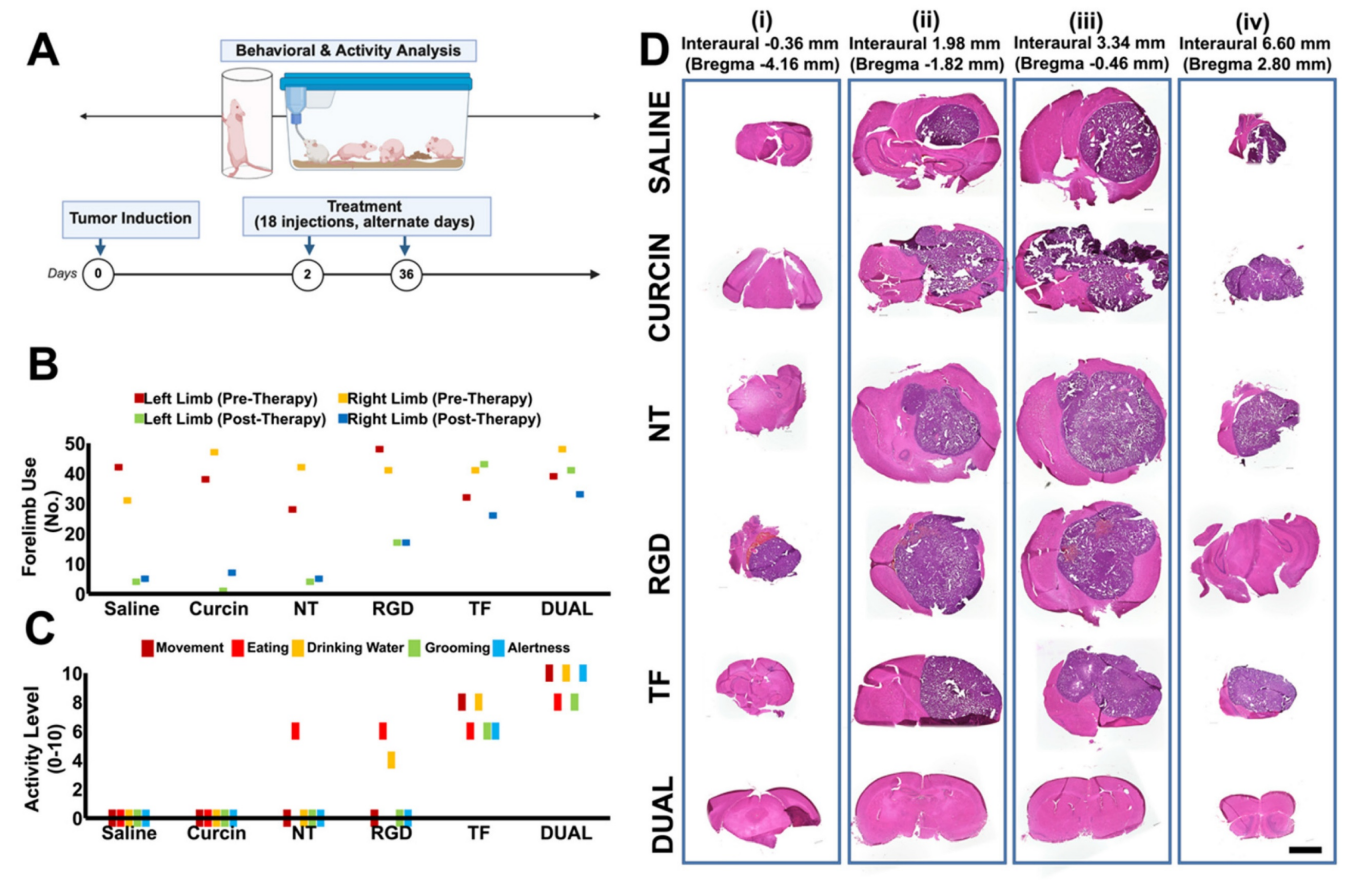
** Dual-HSLNs preserve neurological function and prevent behavioral deficits in gliosarcoma-bearing mice. (A)** Schematic representation of the neurobehavioral study design. Mice underwent tumor induction, followed by 18 curcin or HSLN treatments over 36 days, and subsequent assessment of behavior and brain pathology. **(B)** Quantification of forelimb use (left and right) pre- and post-therapy. Dual-HSLN-treated mice retained bilateral limb symmetry and motor coordination, while saline and NT groups showed progressive asymmetry. **(C)** Behavioral activity scores across five parameters, movement, eating, drinking, grooming, and alertness, on a 0-10 scale. Dual-treated mice exhibited preserved function across all categories; saline and NT groups displayed hypoactivity and behavioral suppression. **(D)** Serial coronal brain sections stained with H&E at four defined interaural/bregma coordinates. Extensive tumor infiltration was evident in saline, NT, and RGD groups, while Dual-HSLN-treated brains showed no visible tumor burden or disruption in functional brain regions. Scale bar = 1 mm. Data in **(B-C)** are presented as individual values or means ± SD (n = 5 per group). Brain regions mapped according to the Allen Mouse Brain Atlas. These results establish that Dual-HSLNs not only eliminate tumors but also preserve sensorimotor integrity and spontaneous behavior, in stark contrast to conventional and monoligand formulations that fail to prevent functional deterioration.

Behavioral phenotyping revealed that Dual HSLN-treated mice retained normal functional parameters throughout the study. Quantitative assessments of exploratory locomotion, limb symmetry, feeding behavior, and sensory reflexes showed no significant deviations from baseline or saline controls **(Figure 6A-C; Table S5)**. Importantly, these animals exhibited no signs of thigmotaxis, a behavioral manifestation of anxiety or sensorimotor dysfunction characterized by wall-hugging behavior during exploration, which was prominently observed in tumor-bearing control groups.

In contrast, saline- and NT HSLN-treated animals exhibited progressive neurological deficits beginning in the second week post-implantation, including pronounced thigmotaxis, forelimb bias, hypoactivity, delayed feeding, and reduced tactile responsiveness, each consistent with tumor-related disruption of motor and cognitive circuits.

To anatomically correlate these symptoms, tumor locations **(Figure 6D)** were mapped to a standardized mouse brain atlas. In saline and NT groups, tumor invasion commonly affected the putamen, corpus callosum, and anterior cingulate cortex, regions essential for motor coordination, interhemispheric communication, and motivational control **(Table S6)**. These patterns were in strong agreement with the observed behavioral impairments. Conversely, Dual HSLN-treated brains exhibited either no visible tumor or confined signal remnants restricted to functionally non-critical areas.

These results emphasize that Dual HSLN therapy not only leads to substantial tumor regression but also preserves neurological function. This multifactorial benefit contrasts with many conventional therapies that, while suppressing tumor burden, frequently introduce or fail to mitigate behavioral toxicity 87, 88.

Previous GBM and GSM models have infrequently evaluated neurobehavioral metrics in conjunction with tumor suppression, despite growing recognition of functional quality of life as a central clinical endpoint 89. Studies utilizing TMZ, radiation, or targeted nanoparticles often demonstrate moderate tumor control but fail to account for cognitive or sensorimotor decline 90, 91. Additionally, nanocarriers bearing cytotoxins or imaging agents may induce unintended neurotoxicity even when effective in reducing tumor size 92, 93. Against this backdrop, the present Dual HSLN formulation presents a notable advance: achieving complete or near-complete tumor clearance while preserving exploratory behavior, sensorimotor integrity, and reflex responsiveness.

This correlation between intact neuroanatomy and preserved function, as supported by brain atlas-based lesion mapping and behavioral scoring, enhances the translational credibility of this platform. To our knowledge, few prior studies have demonstrated such simultaneous tumor eradication and functional protection in GSM models. These findings underline the importance of integrating behavioral endpoints into preclinical evaluations and position Dual HSLNs as a therapeutically comprehensive candidate in CNS oncology research.

Altogether, the convergence of histological, volumetric, and behavioral data provides a robust and multidimensional validation of therapeutic efficacy, aligning with emerging translational standards for preclinical CNS tumor therapies.

### Biocompatibility post-therapy

**Figure 7:**
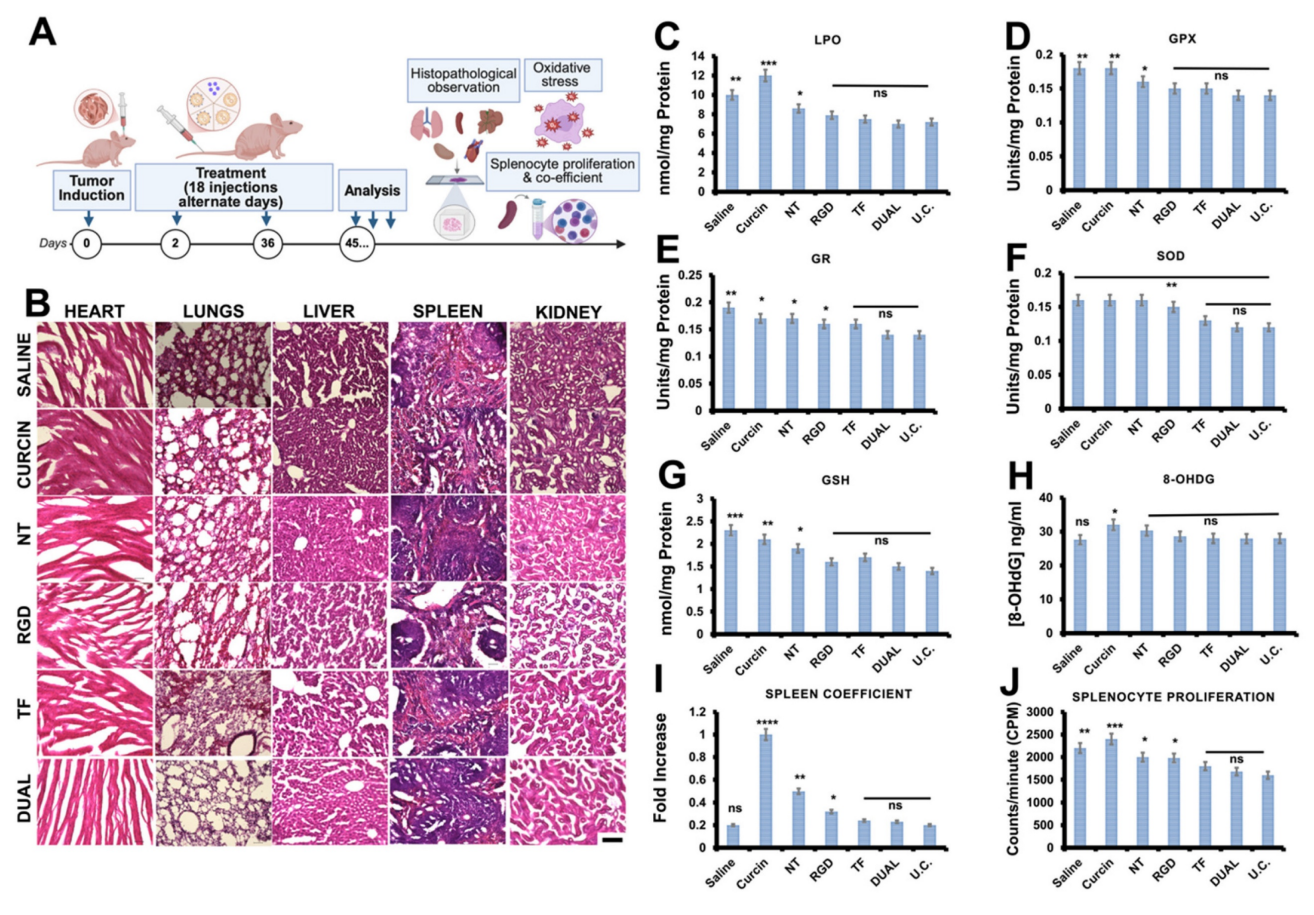
** Systemic biocompatibility, oxidative stress, and immunological safety profile of Dual HSLNs. (A)** Experimental design for post-treatment toxicity and immune profiling. BALB/c-nu mice were evaluated on day 45 after 18 injections of curcin or HSLN formulations. **(B)** Representative H&E-stained sections of heart, lung, liver, spleen, and kidney from each group. All HSLN-treated groups, including Dual, showed intact histoarchitecture, with no signs of inflammation or cellular degeneration. (scale bar = 200 μm) **(C-G)** Quantification of oxidative stress and redox balance markers: **(C)** lipid peroxidation (LPO), **(D)** glutathione peroxidase (GPX), **(E)** glutathione reductase (GR), **(F)** superoxide dismutase (SOD), and **(G)** reduced glutathione (GSH). Dual-HSLNs preserved near-baseline levels of all markers, while free curcin and NT groups exhibited significant redox imbalance. **(H)** Quantification of 8-hydroxy-2′-deoxyguanosine, a biomarker of oxidative DNA damage, revealed genotoxicity in NT and curcin groups but not in Dual. **(I)** Spleen coefficient (organ-to-body weight ratio) as a marker of systemic immune activation. Free curcin markedly increased this coefficient; Dual remained comparable to controls. **(J)** Splenocyte proliferation assay (counts/minute) demonstrating minimal lymphoproliferative stimulation in Dual-HSLN group versus elevated response in free curcin and NT groups. Data in **(C-J)** are presented as mean ± SD (n = 5 mice/group). Statistical analysis: one-way ANOVA with Tukey's post hoc test; ns = not significant, *p* < 0.05 (*), p < 0.01 (**), p < 0.001 (***), p < 0.0001 (****). These results confirm the superior systemic biocompatibility of Dual HSLNs, highlighting their non-toxic, immunologically silent, and redox-stable nature during chronic administration.

To comprehensively evaluate systemic safety and off-target immunotoxicity post-treatment, we analyzed major oxidative stress markers, genotoxicity indices, and spleen-related immune responses in GSM-bearing mice following systemic administration of saline, free curcin, and the various HSLN formulations (NT, RGD, TF, Dual). These assessments were performed at the end of the therapeutic window in the BALB/c-nu model and reflect cumulative systemic effects **(Figure 7A)**.

Histopathological analysis of major organs confirmed the absence of cellular degeneration, inflammation, or histoarchitectural disruption across all treatment groups receiving HSLNs, reaffirming the structural safety of the nanocarriers 82, 94. Notably, the Dual HSLN group displayed organ histology indistinguishable from the saline-treated controls **(Figure 7B)**.

Quantitative assays of antioxidant biomarkers revealed minimal signs of oxidative stress in the Dual HSLN group. Levels of LPO remained low, while the enzymatic activity of GPX, GR, and SOD was preserved near baseline **(Figure 7C-F)**. Additionally, reduced GSH levels were well maintained **(Figure 7G)**. Importantly, the DNA oxidation biomarker 8-OHdG was undetectable in brain tissue, confirming the absence of genotoxicity **(Figure 7H)** 95, 96. These parameters were slightly elevated in the TF and RGD single-ligand groups but remained within tolerable bounds.

The spleen coefficient and splenocyte proliferation assays served as proxies for systemic immune activation and lymphoid hyperplasia. Both measures were equivalent between the Dual HSLN and control groups, suggesting that the formulation does not provoke an adverse immune response **(Figure 7I-J)**. In contrast, animals treated with free curcin exhibited significantly elevated spleen coefficients, highlighting the lymphoproliferative toxicity associated with systemic exposure to unencapsulated RIPs 97, 98.

These observations align with recent reports in GBM and GSM nanomedicine, where lipidic or PEGylated systems have shown better systemic compatibility relative to polymeric micelles or dendrimeric constructs. For instance, PEGylated liposomes and solid lipid nanoparticles delivering doxorubicin or paclitaxel have demonstrated moderate improvements in systemic oxidative stress and splenic indices post-treatment 99, 100. However, those carriers often fall short in achieving the levels of redox normalization or genotoxic protection observed in the Dual HSLNs presented here.

Moreover, studies involving polymer-based or silica nanocarriers, while effective in tumor regression, frequently trigger elevated LPO or GSH depletion in hepatic tissues at comparable doses 101, 102. The superior redox and immunological safety profile seen with Dual HSLNs likely stems from the combined effect of their biocompatible lipid matrix, surface PEGylation, and controlled, tumor-restricted curcin release.

These findings confirm the translational safety of the Dual HSLNs for CNS tumor therapy, demonstrating not only histological and hematological normalcy, but also genomic and immunological safety under chronic administration protocols.

### Computational docking studies

To elucidate the molecular mechanisms underlying curcin's anti-tumor efficacy, computational docking studies were conducted against four GSM-associated receptors known to regulate tumor proliferation, angiogenesis, and immune evasion: epidermal growth factor receptor (EGFR), ephrin type-A receptor 2 (EphA2), interleukin-13 receptor alpha 2 (IL-13Rα2), and metabotropic glutamate receptor 6 (mGluR6). These receptors are commonly overexpressed in GSM tissues and have been implicated in aggressive phenotypes and therapeutic resistance 78, 103.

Molecular docking simulations were performed using the HDOCK platform (v.2023-09-09) 104, integrating homology modeling and ab initio structure prediction. Both the ligand-binding domains (LBDs) and intracellular domains (ICDs) of the receptor proteins were targeted to investigate potential for dual inhibition, namely, extracellular ligand competition and intracellular signaling interference **(Figure S13)**. Curcin was docked against the LBDs of EGFR (PDB: 1IVO), EphA2 (PDB: 2X10), IL-13Rα2 (PDB: 3LB6), and the AlphaFold-predicted structure of mGluR6 (AF-O15303).

The predicted binding energies ranged from -9.3 to -11.6 kcal/mol, indicating strong thermodynamic favorability and high-affinity interactions. Notably, curcin showed robust docking scores for both the LBD and ICD of all four receptors. These interactions suggest that curcin may function via a bifunctional inhibitory mechanism: competitively blocking receptor-ligand binding and simultaneously preventing receptor dimerization or kinase activation at the cytoplasmic interface. Such dual inhibition may contribute to broader suppression of proliferative and angiogenic signaling cascades in GSM.

Compared to conventional small-molecule inhibitors or monovalent biologics, which typically bind a single domain on a single receptor, curcin's multi-receptor and multi-domain affinity presents a critical advantage in combating intratumoral heterogeneity, a hallmark of GSM. This aligns with its observed *in vivo* performance, particularly when delivered via Dual HSLNs, which enable spatially targeted accumulation in the tumor microenvironment.

Furthermore, curcin's binding configuration supports its classification as a “bifunctional” anticancer agent, consistent with recent strategies employing multi-site targeting to improve efficacy in resistant tumor models. These bifunctional agents are increasingly recognized for their ability to overcome escape mechanisms and produce more durable therapeutic responses 105, 106.

Together, these *in silico* results provide molecular-level validation of curcin's polypharmacological action and reinforce the rationale for its integration within dual-targeted HSLNs. The observed receptor interactions, particularly the simultaneous engagement of EGFR, EphA2, IL-13Rα2, and mGluR6, may underpin the therapeutic synergy observed in preclinical models and warrant further investigation through structural and cellular biology approaches.

In conclusion, this study presents a robust and translationally promising dual-ligand nanoplatform that combines integrin (RGD) and TF targeting for efficient curcin delivery to GSM tumors. By enabling synergistic receptor-mediated transcytosis across the BBB and enhanced tumor retention, the Dual HSLNs achieved superior therapeutic accumulation and complete regression in the majority of treated animals. This targeted delivery system not only disrupted neo-angiogenesis and oncoprotein synthesis via curcin's multimodal cytotoxic abilities, including protein synthesis inhibition, but also preserved neurological function—a key yet often overlooked clinical outcome. Biodistribution, redox profiling, and behavioral assays collectively validated the long-term biocompatibility of the formulation, while molecular docking confirmed strong, bifunctional interactions of curcin with key GSM-relevant receptors (EGFR, EphA2, IL-13Rα2, and mGluR6), offering polypharmacological breadth beyond conventional approaches.

Importantly, GSM remains severely underrepresented in nanotherapy research, with most efforts focused on GBMs despite GSM's heightened aggressiveness and therapeutic resistance. To our knowledge, this is among the first studies to demonstrate durable tumor regression, functional brain protection, and receptor-level mechanistic validation in a GSM-targeted preclinical model. While a fraction of animals exhibited residual tumors, the observed reductions in tumor volume and extensions in survival strongly support the platform's therapeutic promise.

Future studies should explore this system's application in late-stage or non-resectable GSM models and assess alternative or combinatorial payloads, such as gene editing tools, delivered via this platform to overcome current CNS delivery bottlenecks. Because the carrier lipids (DSPE-PEG, stearic acid, lecithin) and ligands (TF, RGD) are all GRAS-listed or previously used in clinical formulations, the platform is readily scalable for GMP manufacture. Altogether, the RGD-TF Dual HSLN system offers a multifunctional and translationally adaptable strategy not only for GSM, but also for other high-grade gliomas and CNS tumors characterized by BBB heterogeneity, while simultaneously establishing a clinically relevant delivery platform for RIPs like curcin, a class of potent yet underutilized anticancer agents in neuro-oncology.

## Supplementary Material

Supplementary figures and tables. The supporting information file contains additional experimental data.

## Data Availability

The data that support the findings of this study are available from the corresponding author upon reasonable request.

## Abbreviations

BBB: blood-brain barrier
CNS: central nervous system
EPR: enhanced permeability and retention
GBM: glioblastoma multiforme
GSM: gliosarcoma
HSLN: hybrid solid lipid nanoparticle
NT: non-targeted
RES: reticuloendothelial system
RIPs: ribosome-inactivating proteins
TEER: transendothelial electrical resistance
TF: transferrin
TfR: transferrin receptor
