# Supplementary material for: Dual-ligand curcin-loaded hybrid solid lipid nanoparticles achieve durable gliosarcoma remission while preserving neuro-behavioral function
Source: Theranostics. 2026 Mar 4;16(10):5150–74. doi: 10.7150/thno.123534 (PMC13080334; doi:10.7150/thno.123534)
Supplement: Supplementary file 1 — Supplementary figures and tables. [file thnov16p5150s1.pdf]

Dual-ligand curcin-loaded hybrid solid lipid nanoparticles achieve durable gliosarcoma remission while preserving neuro-behavioral function

Mohamed Sheikh Mohamed[1,2#], Srivani Veeranarayanan[1,3], Yasushi Sakamoto[4#], Rie Suge[5], Narumi Hirosawa[4], Aby Cheruvathoor Poulose[1,6], Toru Mizuki[1,2], Toru Maekawa[1,2]

[1]*Bio-Nano Electronics Research Centre, Toyo University, Kawagoe, Saitama, 350-8585, Japan.*

[2]*Graduate School of Interdisciplinary New Science, Toyo University, Kawagoe, Saitama, 350-8585, Japan.*

[3]*Division of Bacteriology, Department of Infection & Immunity, Jichi Medical University Shimotsuke-Shi, Tochigi, 329-0498, Japan.*

[4]*Biomedical Research Centre, Division of Analytical Science, Saitama Medical University, Saitama 350-0495, Japan.*

[5]*Department of Liberal Arts, Saitama Medical University, Saitama 350-0495, Japan.*

[6]*Regional Centre of Advanced Technologies and Materials, Czech Advanced Technology and Research Institute (CATRIN), Palacký University in Olomouc, Šlechtitelů 27, 783 71, Olomouc, Czech Republic*

SUPPLEMENTARY INFORMATION (SI) FIGURES

# Corresponding authors

(sheikh@toyo.jp & ysakamot@saitama-med.ac.jp)

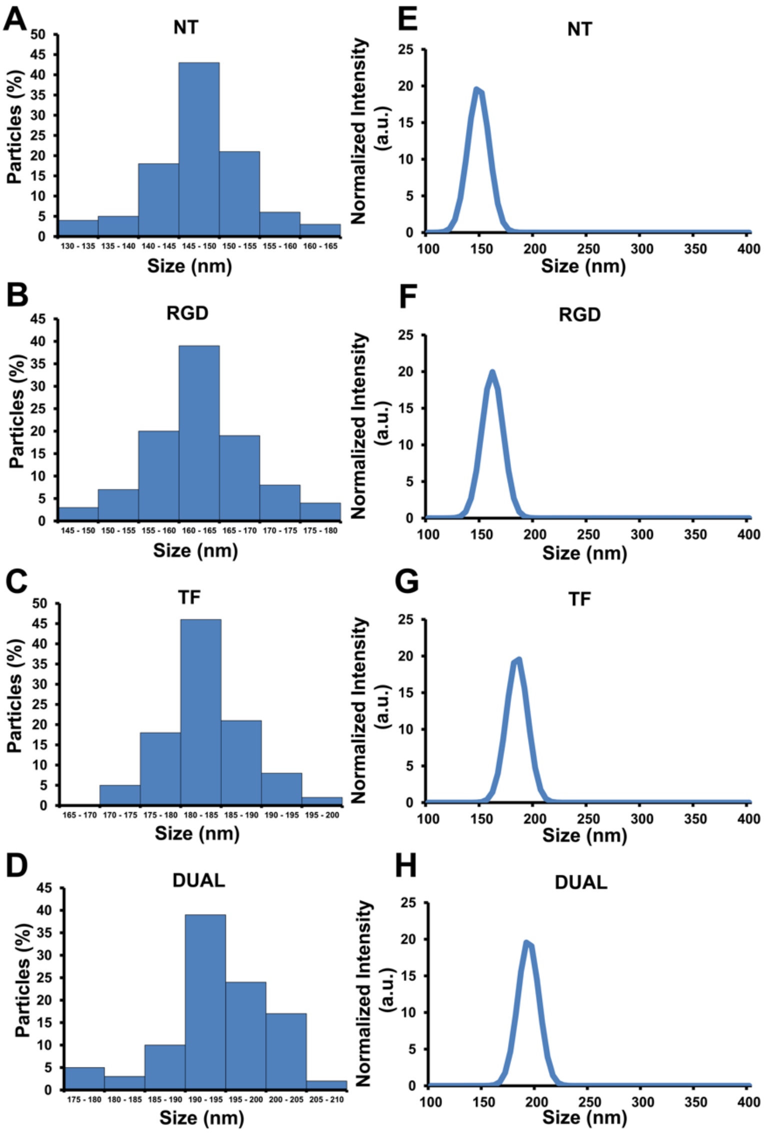

Figure S1. Particle size characterization of functionalized HSLNs.
Left panels show transmission electron microscopy (TEM)–based particle size histograms obtained by manual measurement of 100 individual nanoparticles for each formulation: **(A)** non-targeted (NT), **(B)** RGD-functionalized, **(C)** transferrin (TF)-functionalized, and **(D)** DUAL-functionalized (RGD+TF) HSLNs. Right panels **(E-H)** show the corresponding dynamic light scattering (DLS)–derived hydrodynamic size distributions (intensity-weighted, normalized intensity in arbitrary units). All formulations exhibited monomodal DLS profiles with low polydispersity (PDI < 0.3), confirming homogeneous nanoparticle populations. NT and RGD HSLNs displayed smaller mean diameters (~147 nm and ~160 nm, respectively), consistent with sizes favorable for enhanced permeability and retention (EPR) and αvβ3 integrin-mediated uptake. In contrast, TF (~183 nm) and dual-functionalized (~192 nm) HSLNs showed larger hydrodynamic sizes, attributable to increased surface ligand density and higher molecular-weight conjugation via NHS–EDC chemistry.

Table S1. Stability of HSLNs. Notably, the formulations were stable for up to 6 months in suspension and more than 1 year in pellet form, with no particle aggregation or significant change in hydrodynamic diameter and zeta potential (196 ± 30.0 nm with potential of −12 ± 2.1 mV), which could be explained by the PEG bound to the NP surface, providing a hydrophilic steric barrier that prevents particle aggregation. Additionally, the chemical stability of HSLNs was determined by incubating them in 50% FBS. Post 24 h, the hydrodynamic diameter and zeta potential were assessed and found to be similar to the FBS-untreated HSLNs (194 ± 30.0 nm and −10 ± 1.8 mV) depicting the NPs resistance to serum degradation and protein corona formation, eventually solidifying their applicability for systemic injection in preclinical applications. Data are reported as mean ± standard deviation (SD) from three independent measurements (n = 3).

Conditioning Medium	Size (Hydrodynamic)	Zeta Potential
Fresh Suspension	192 ± 20.0 nm (PDI < 0.3)	−8 ± 1.5 mV
PBS Suspension (post 6 months)	196 ± 30.0 nm (PDI < 0.4)	−12 ± 2.1 mV
50% fetal bovine serum (post 24 h)	194 ± 30.0 nm (PDI < 0.5)	−10 ± 1.8 mV

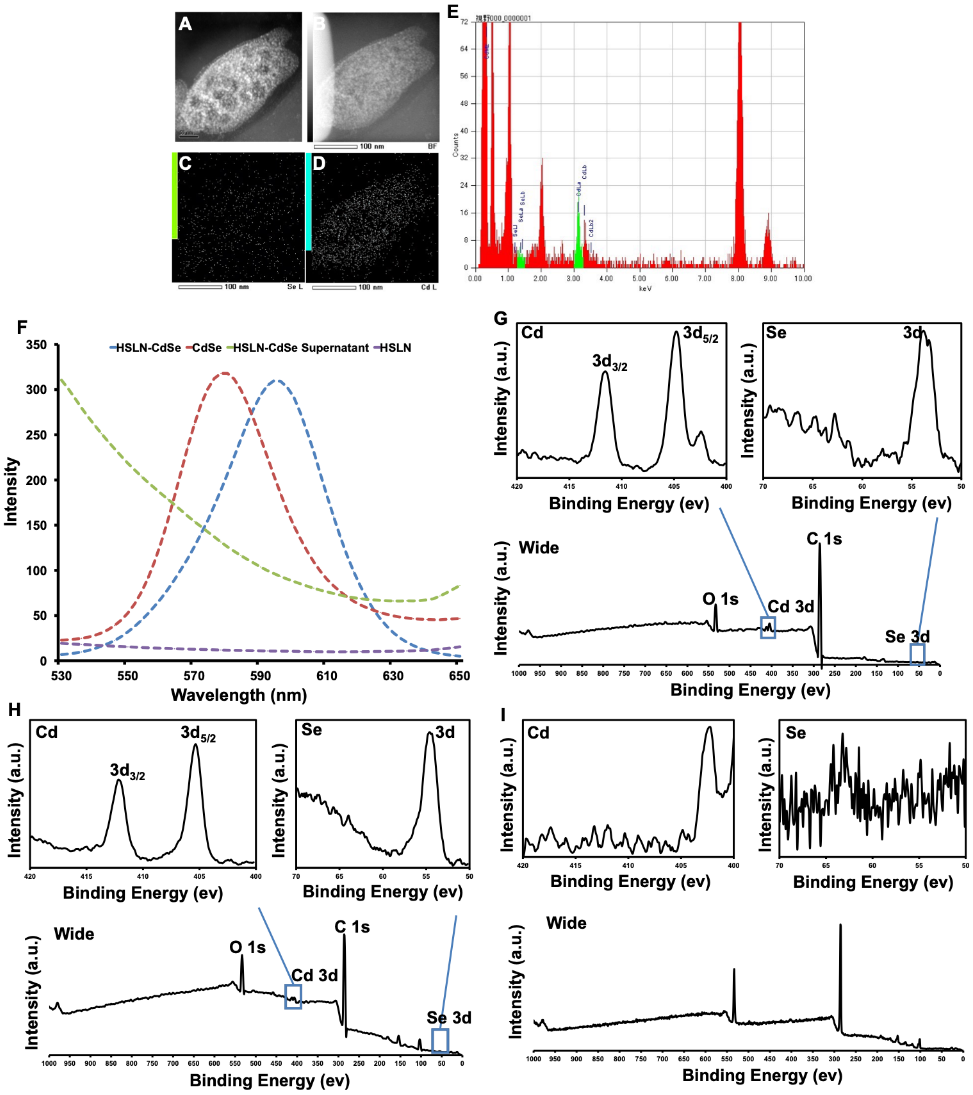

Figure S2. Structural, optical, and elemental characterization of CdSe-embedded HSLNs (HSLN–CdSe). (A-B) TEM images of HSLN–CdSe nanoparticles revealing preserved morphology post QD encapsulation. Uniform contrast indicates homogeneous CdSe loading without aggregation or shape deformation. (scale bar = 100 nm) **(C-D)** Elemental mapping for Se and Cd, confirming even QD distribution within the lipid matrix. Elemental dispersion is spatially confined within nanoparticle boundaries, suggesting stable encapsulation.
(E) EDX of HSLN–CdSe confirms strong signals corresponding to Cd and Se.
(F) PL emission spectra of HSLN–CdSe vs. free CdSe QDs and controls. Encapsulated QDs maintained their characteristic emission peak (~570 nm), with a slight red-shift (peak maxima ~600 nm), while the supernatant and empty HSLNs showed negligible background. This result confirms photostability and integrity of QD structure post-encapsulation.
(G-I) XPS analyses comparing **(G)** HSLN–CdSe, **(H)** free CdSe QDs, and **(I)** blank HSLNs. HSLN–CdSe spectra showed strong Cd 3d ($3d_{5/2}$ and $3d_{3/2}$) and Se 3d peaks with minimal surface oxidation, confirming intact QD structure. The absence of free Cd^{2+} or Se^0 species in the wide-scan spectra suggests negligible cadmium leaching. These findings directly support the *in vivo* biocompatibility and diagnostic applicability discussed in the Results & Discussion section. All measurements were conducted using triplicate samples to confirm reproducibility.

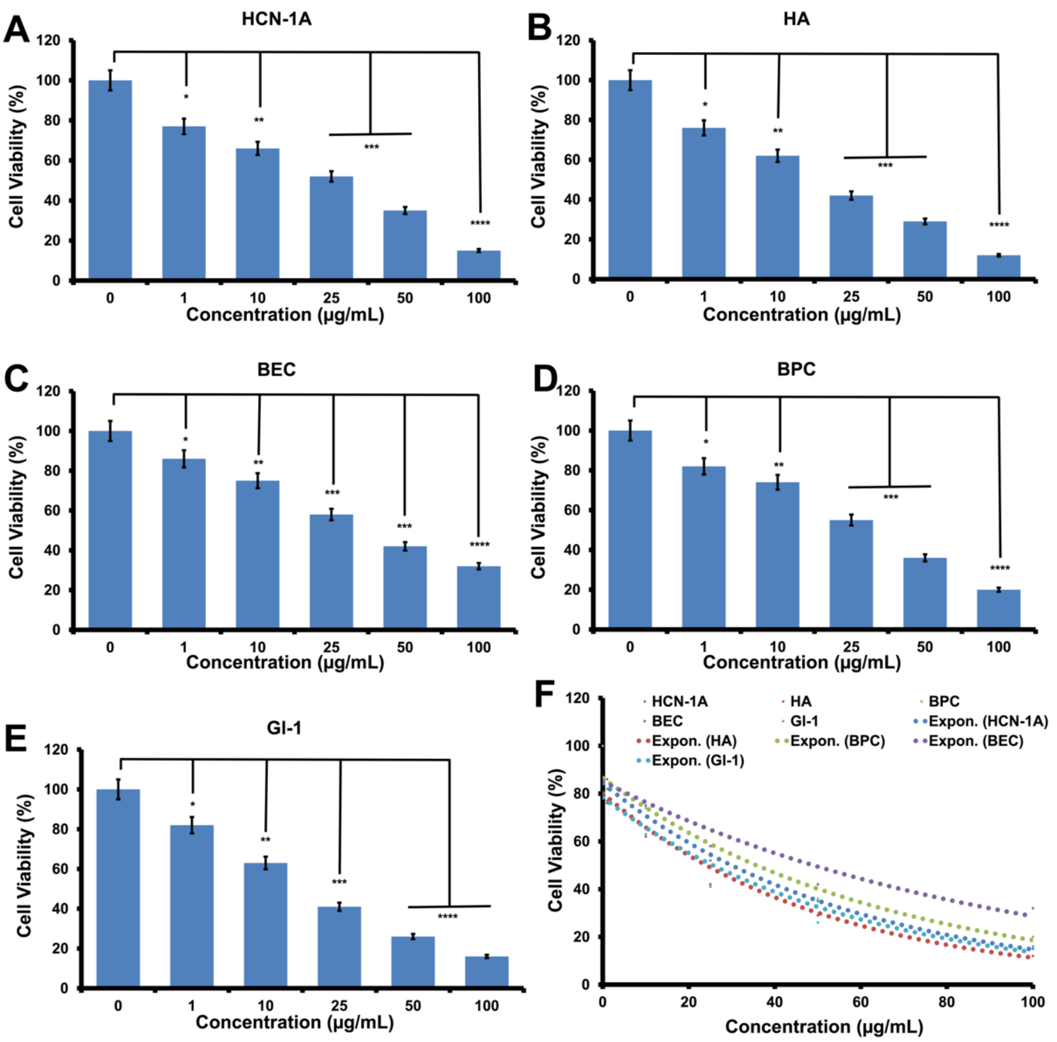

Figure S3. Dose-dependent cytotoxicity profile of free curcin across CNS and gliosarcoma-relevant cell types (A-E) Cell viability of **(A)** human cortical neurons (HCN-1A), **(B)** human astrocytes (HA), **(C)** human brain endothelial cells (BEC), **(D)** human brain pericytes (BPC), and **(E)** human GI-1 gliosarcoma cells treated with increasing concentrations of free curcin (0–100 µg/mL) for 72 h. Viability was measured using Alamar Blue assay, normalized to untreated controls, and plotted as mean ± standard deviation (n = 3). A clear concentration-dependent cytotoxic response was observed in all cell types, with GI-1, HA & HCN-1A exhibiting the highest sensitivity. This trend is consistent with curcin's mechanism as a ribosome-inactivating protein (RIP), which depurinates 28S rRNA and inhibits protein synthesis. **(F)** Exponential regression curves of cell viability trends with corresponding IC_{50} values: GI-1 (13.5 µg/mL), HCN-1A (16.1 µg/mL), HA (17.8 µg/mL), BEC (18.9 µg/mL), and BPC (22.9 µg/mL). Statistical analysis: one-way ANOVA with Tukey's post hoc test; ns = not significant, $p < 0.05$ (*), *p < 0.01 (**), p < 0.001 (****), *p < 0.0001 (****)*. These results confirm that free curcin exerts broad-spectrum cytotoxicity, with little discrimination between malignant and non-malignant CNS cells. This underscores the necessity for targeted delivery systems to mitigate off-target neurotoxicity, an objective addressed by the curcin-loaded dual-targeted HSLNs developed in this study. The observed toxicity in BEC and BPC also supports the finding that free curcin compromises BBB integrity, as evidenced by TEER decline **(Figure S5)**, whereas HSLN encapsulation preserves endothelial viability. All cytotoxicity assays were performed in triplicate.

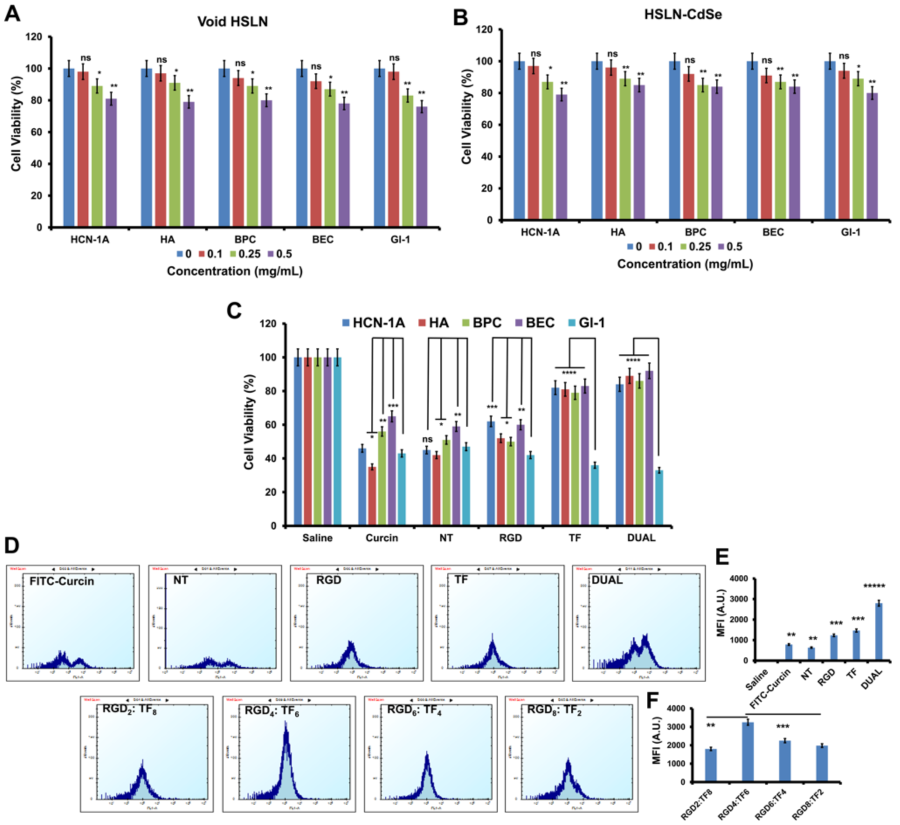

Figure S4. Cytocompatibility of HSLNs and gliosarcoma-specific cytotoxicity of curcin-loaded formulations. (A-B) Cell viability of HCN-1A, HA, BPC, BEC, and GI-1 cells after 72 h treatment with **(A)** void HSLNs (devoid of curcin) and **(B)** HSLNs encapsulating CdSe quantum dots (HSLN–CdSe) (devoid of curcin) at 0.1–0.5 mg/mL. Cell viability was measured using Alamar Blue assay. Both formulations exhibited high cytocompatibility across all cell types, with viability ≥ 90% at all tested doses. No significant toxicity was observed in normal CNS-associated cells or GI-1 tumor cells. **(C)** Comparative cytotoxicity of free curcin, NT, single-ligand (RGD or TF), and dual-ligand (RGD+TF) curcin-loaded HSLNs across five cell types. Free curcin, NT and RGD variants exhibited commendable cytotoxicity to GI-1 cells, however it was marred by the non-specific toxicity observed in the normal cells as well. TF and Dual on the other hand, showed enhanced selectivity for GI-1 over non-malignant cells. These results reflect ligand-specific targeting via transferrin receptors (TfR) and integrins (αvβ3/α5β1), both overexpressed in gliosarcoma. For each cell line, saline-treated controls were used as the internal reference and normalized to 100%; all treatment groups are expressed as % of the corresponding cell-line control (mean ± SD, n = 3). **(D)** Flow cytometry-based quantification of cellular uptake of curcin-FITC or HSLN-CdSe formulations in GI-1 cells and **(E-F)** respective mean fluorescent intensity graph. Among all groups, Dual HSLNs with a 4:6 RGD:TF molar ratio (RGD_4:TF_6) achieved the highest fluorescence intensity, indicating superior internalization. Both under- and over-modified ligand ratios (e.g., RGD_6:TF_2 and RGD_2:TF_8) resulted in a comparative decrease in uptake, supporting the hypothesis that ligand stoichiometry critically influences receptor-mediated endocytosis and cellular binding avidity. Data in **(A-C, E-F)** are presented as mean ± SD (n = 3). Statistical analysis: one-way ANOVA with Tukey's post hoc test; ns = not significant, $p < 0.05$ (*), $p < 0.01$ (**), $p < 0.001$ (***), $p < 0.0001$ (****). These findings confirm that (i) HSLN-CdSe formulations are intrinsically biocompatible, (ii) dual-ligand targeting enhances curcin selectivity toward gliosarcoma cells, and (iii) optimal ligand balance (4:6 RGD:TF) maximizes cellular uptake.

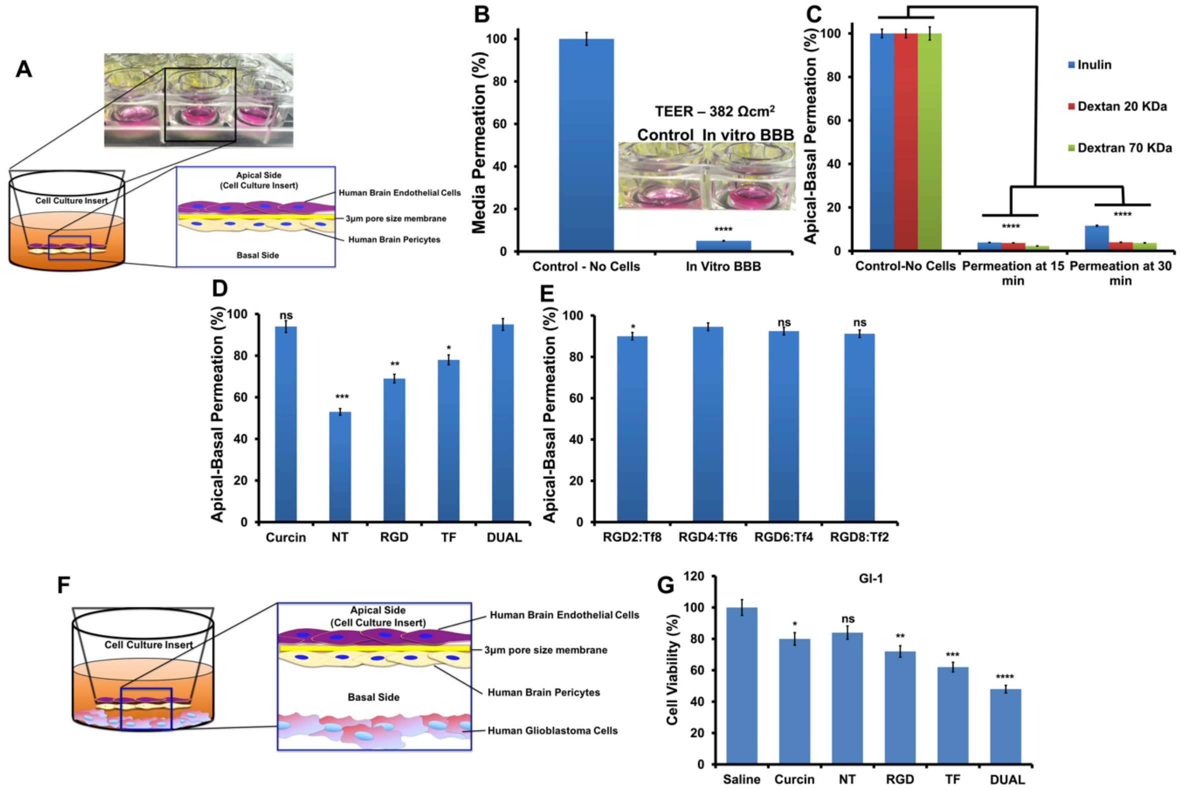

Figure S5. *In vitro* BBB transport validation and post-transcytosis cytotoxicity of curcin-loaded HSLNs (A) Schematic representation of the *in vitro* BBB model comprising BEC on the apical insert membrane and human brain pericytes BPC on the basal surface. A tight junction monolayer mimicking the physiological BBB was established over 4–5 days. **(B)** Quantification of media permeation across inserts lacking cells (control) versus inserts containing the co-culture BBB model. TEER values exceeding 380 $\Omega \cdot cm^2$ confirmed tight junction formation and barrier integrity.
(C) Apical-to-basal permeation of paracellular transport tracers, Inulin, Dextran 20 kDa, and Dextran 70 kDa, at 15 and 30 min. Minimal tracer passage across the *in vitro* BBB validates the model's selective permeability. **(D)** Apical-to-basal translocation efficiency of curcin-FITC or curcin-loaded HSLNs (NT, RGD, TF, Dual) across the *in vitro* BBB after 2 h exposure. Dual-targeted HSLNs demonstrated the highest permeation (~95%), outperforming single-ligand and non-targeted systems.
(E) Permeation profiles of ligand stoichiometry variants RGD_2:TF_8, RGD_4:TF_6, RGD_6:TF_4, and RGD_8:TF_2 confirm that the 4:6 molar ratio achieved optimal BBB transport with minimal variance. These findings support ligand balance optimization to enhance transcytosis via both integrin- and TfR-mediated pathways. **(F)** Schematic of an extended BBB-GSM model with GI-1 gliosarcoma cells seeded in the basal compartment to simulate tumor interface.
(G) Post-transcytosis cytotoxicity in GI-1 cells following 4 h nanoparticle exposure and 72 h incubation. Dual-targeted HSLNs induced the greatest viability reduction, indicating effective curcin delivery through the BBB and release at the tumor interface. TEER values remained stable post-treatment with HSLNs, whereas free curcin disrupted the barrier. Data in **(B-E, G)** as mean ± SD, n = 3; Statistical analysis: one-way ANOVA with Tukey's post hoc test; ns = not significant, $p < 0.05$ (*), $p < 0.01$ (**), $p < 0.001$ (***), $p < 0.0001$ (****). These results demonstrate that (i) the *in vitro* BBB model exhibits selective barrier function, (ii) Dual HSLNs achieve superior translocation and tumor cytotoxicity, and (iii) optimized ligand ratios enhance coordinated receptor-mediated transport. Together, these findings confirm the functional delivery capability of HSLNs and support translational potential for CNS-targeted nanotherapy.

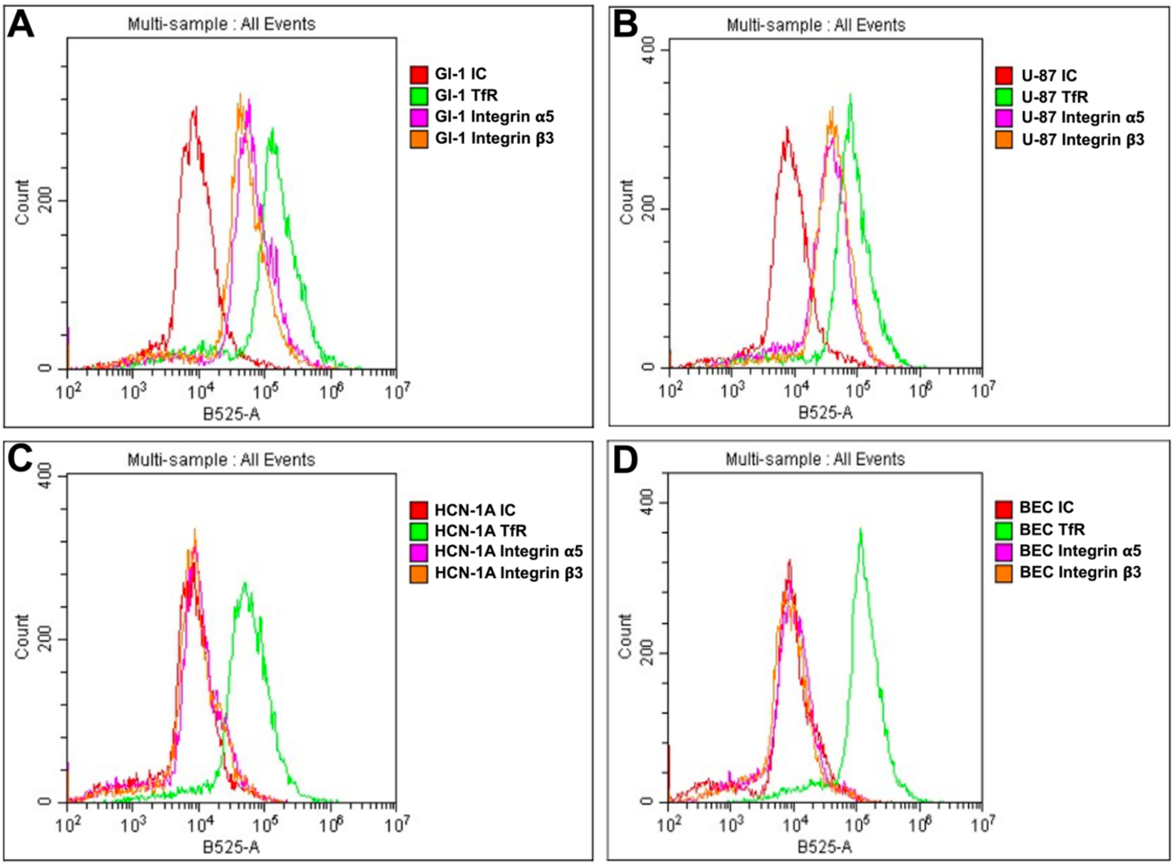

Figure S6. Cell surface expression of transferrin receptor and integrins in brain tumor and non-tumor cell models.
Flow cytometry histogram overlays showing surface expression of transferrin receptor (TfR), integrin α5, and integrin β3 in **(A)** GI-1 gliosarcoma cells, **(B)** U-87MG glioma cells, **(C)** HCN-1A human cortical neurons, and **(D)** brain endothelial cells (BECs). Corresponding isotype controls (IC) are shown for each cell type. GI-1 and U-87 cells exhibited pronounced TfR expression together with detectable integrin α5 and β3 signals relative to isotype controls, whereas HCN-1A cells and BECs displayed clear TfR expression but minimal integrin α5/β3 signals near background levels. These receptor expression profiles support the rationale for dual-ligand targeting, combining TfR-mediated BBB engagement with integrin-mediated tumor cell uptake. The presence of both TfR and integrin receptors on glioma cells provides a mechanistic basis for the observed compensatory uptake behavior under single-receptor blocking conditions.

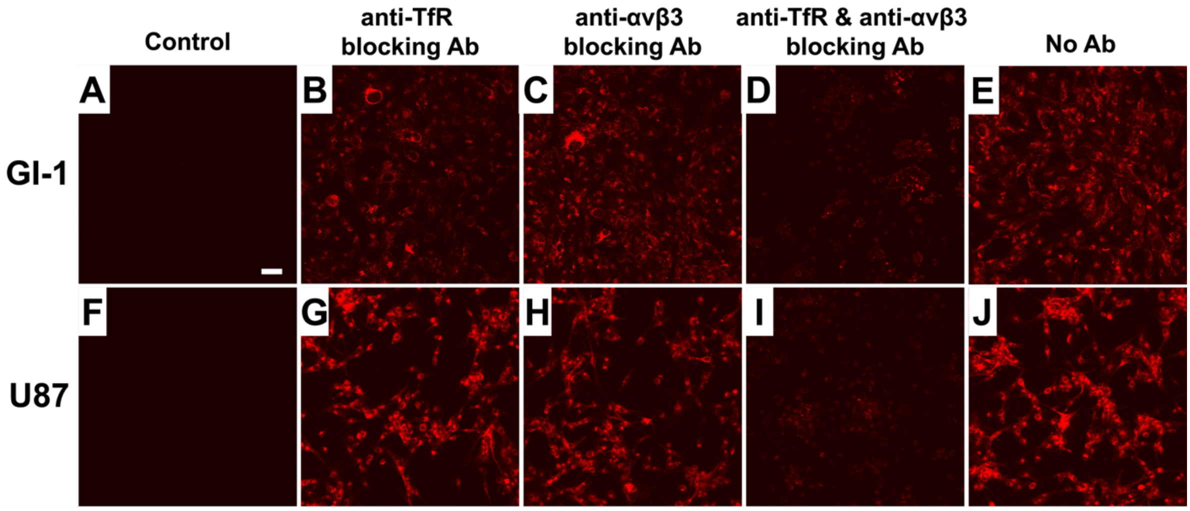

Figure S7. Cooperative contribution of transferrin receptor and integrin pathways to dual-ligand HSLN uptake in brain tumor cells. Representative confocal fluorescence micrographs showing intracellular uptake of fluorescently labeled dual-functionalized (TfR + RGD) HSLNs in **(A-E)** GI-1 gliosarcoma cells and **(F-J)** U-87MG glioma cells following incubation at 37 °C. Panels **(A)** and **(F)** show background fluorescence in cells not exposed to HSLNs. Panels **(B-C)** and **(G-H)** show uptake following single-receptor blocking (anti-TfR or anti-integrin antibody), which did not markedly reduce intracellular fluorescence compared with the no-blocking controls **(E-J)**. In contrast, simultaneous blocking of both TfR and integrin receptors **(D-I)** resulted in a pronounced reduction in intracellular nanoparticle-associated fluorescence. These observations indicate that dual-ligand HSLN uptake is mediated through cooperative and partially compensatory receptor-dependent pathways, rather than reliance on a single transport mechanism. All images were acquired using identical imaging parameters. (Scale bar = 100 μm).

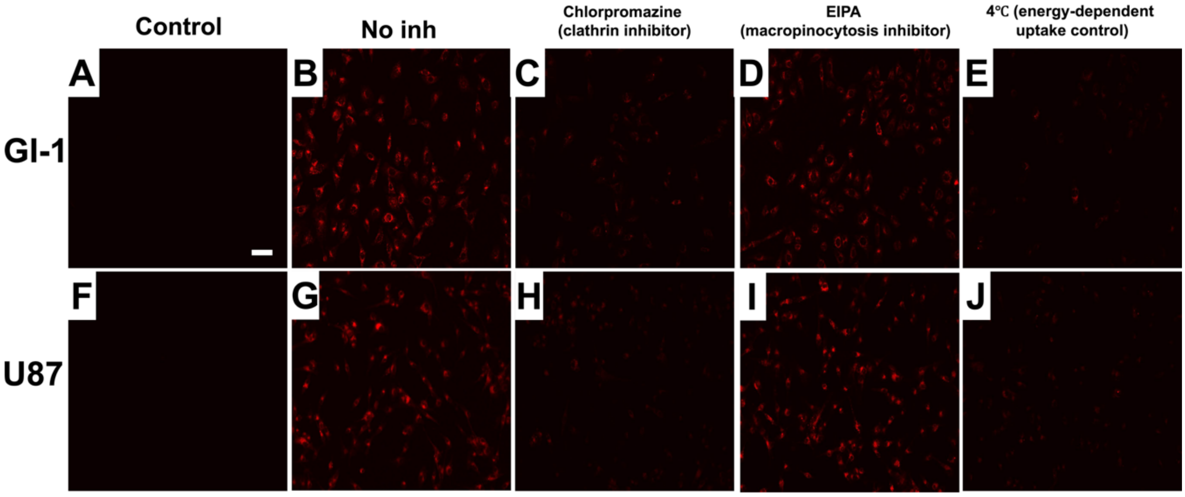

Figure S8. Endocytic pathway involvement in dual-ligand HSLN uptake in glioma cell models. Representative confocal fluorescence micrographs showing intracellular uptake of fluorescently labeled dual-functionalized (TfR + RGD) HSLNs in **(A-E)** GI-1 gliosarcoma cells and **(F-J)** U-87MG glioma cells following pharmacological inhibition of endocytic pathways. Panels **(A)** and **(F)** show background fluorescence in cells not exposed to HSLNs. Panels **(B)** and **(G)** show nanoparticle uptake under vehicle-treated conditions (no inhibitor). Panels **(C)** and **(H)** show uptake following inhibition of clathrin-mediated endocytosis, which resulted in a marked reduction in intracellular fluorescence comparable to that observed under energy-restricted conditions at 4 °C **(E-J)**. In contrast, inhibition of macropinocytosis **(D-I)** did not produce a noticeable reduction in uptake relative to vehicle controls. These observations indicate that internalization of dual-ligand HSLNs is energy-dependent and occurs predominantly via clathrin-mediated endocytic pathways. All images were acquired using identical imaging parameters. (Scale bar = 100 µm).

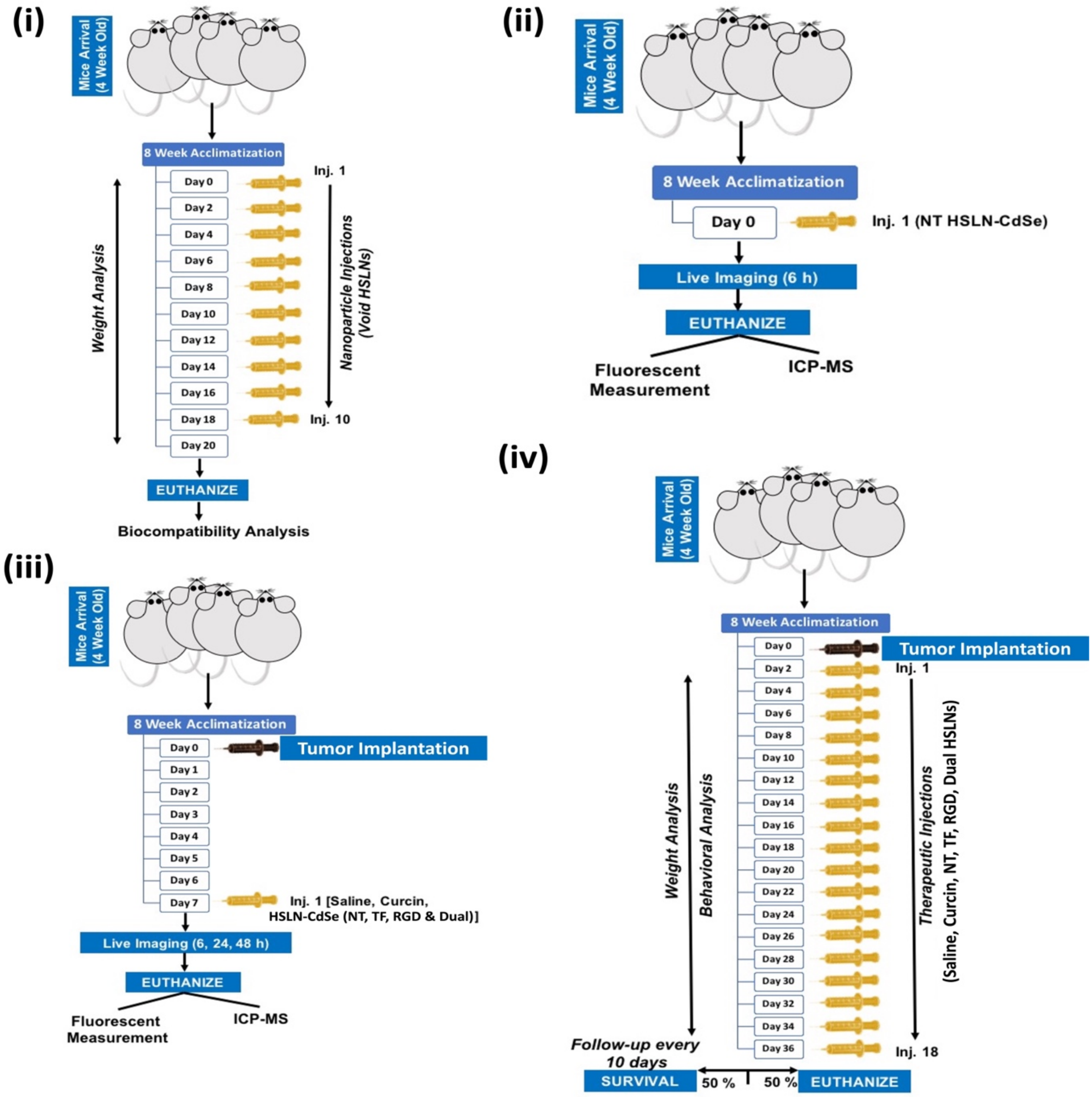

Scheme S1. Overview of *in vivo* experimental design. Four sequential mouse studies were conducted following an 8-week acclimatization: **(i)** systemic safety of void HSLNs by repeated dosing over 20 days; **(ii)** acute biodistribution of CdSe-loaded HSLNs via single-dose fluorescence imaging and ICP-MS; **(iii)** early tumour targeting in GI-1 gliosarcoma–bearing mice with single-dose CdSe-HSLNs (non-targeted, single-ligand, dual-ligand) assessed at 6, 24, and 48 h; and **(iv)** therapeutic efficacy and survival following 18 alternate-day injections of saline, free curcin, or curcin-loaded HSLNs (non-targeted, single-ligand, dual-ligand), with mid-study mechanistic read-outs and Kaplan–Meier analysis to day 80. Continuous monitoring of weight, behavior, and humane-endpoint criteria ensured animal welfare throughout.

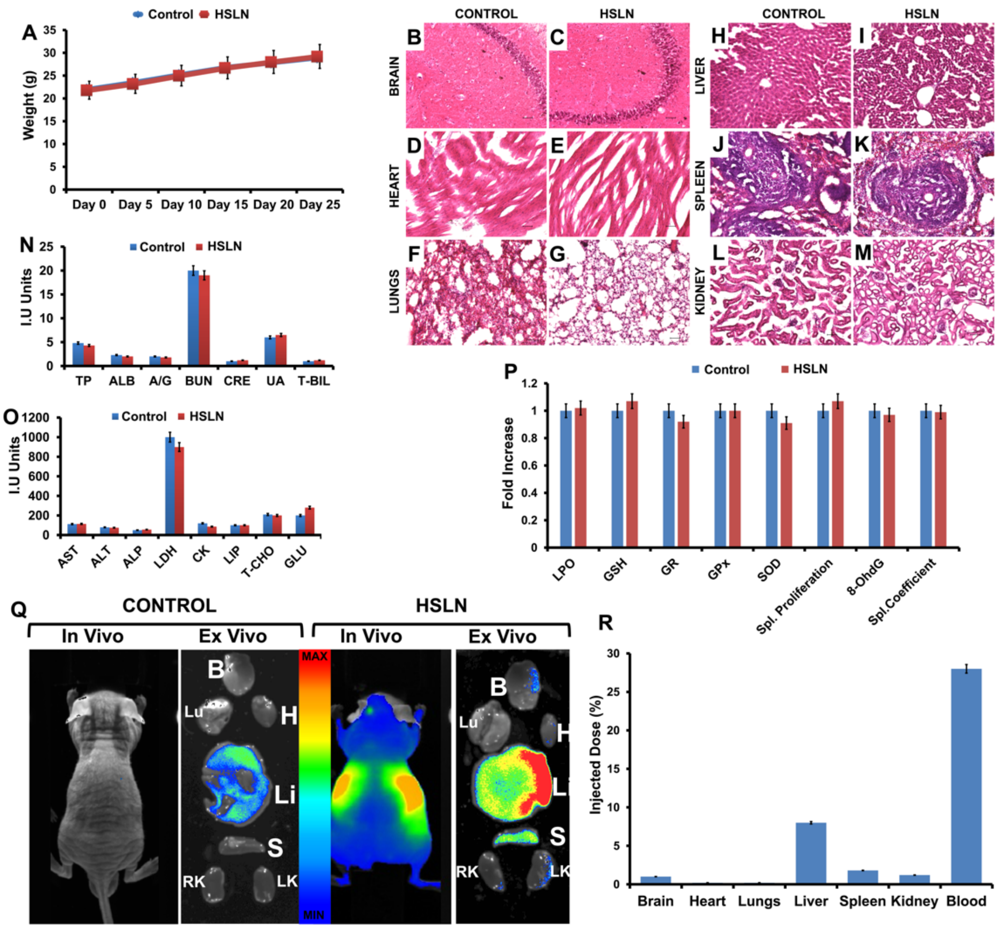

Figure S9. Systemic biocompatibility, biodistribution, and safety profile of void HSLNs in healthy BALB/c-nu mice (A) Body weight tracking over 25 days following intravenous administration of void HSLNs every other day for 20 days (10 doses total, n = 10). No significant difference was observed between HSLN-treated and saline-treated groups, indicating systemic tolerance.
(B-M) Representative H&E-stained sections of brain, heart, lungs, liver, spleen, and kidney show no histopathological alterations in HSLN-treated mice. (Scale bar = 200 µm).
(N-O) Serum biochemical profiles of hepatic and renal function showed no statistically significant changes between HSLN and control groups across all markers (TP, ALB, BUN, CRE, AST, ALT, etc).
(P) Fold change analysis of oxidative stress markers (LPO, GSH, GR, GPX, SOD), genotoxicity (8-OHdG), splenocyte proliferation, and spleen coefficient revealed no statistically significant differences from controls, confirming redox and immune neutrality.
(Q-R) Fluorescence imaging and ICP-MS analysis demonstrate biodistribution of NT HSLN–CdSe nanoparticles. No detectable accumulation was observed in brain tissue, and statistical comparison of organ-specific uptake showed no abnormalities attributable to nanoparticle administration. Data in **(A, N, O, P, R)** are presented as mean ± SD (n =10). Statistical analysis: one-way ANOVA with Tukey's post hoc test; no statistically significant differences observed ($p > 0.05$ for all comparisons).

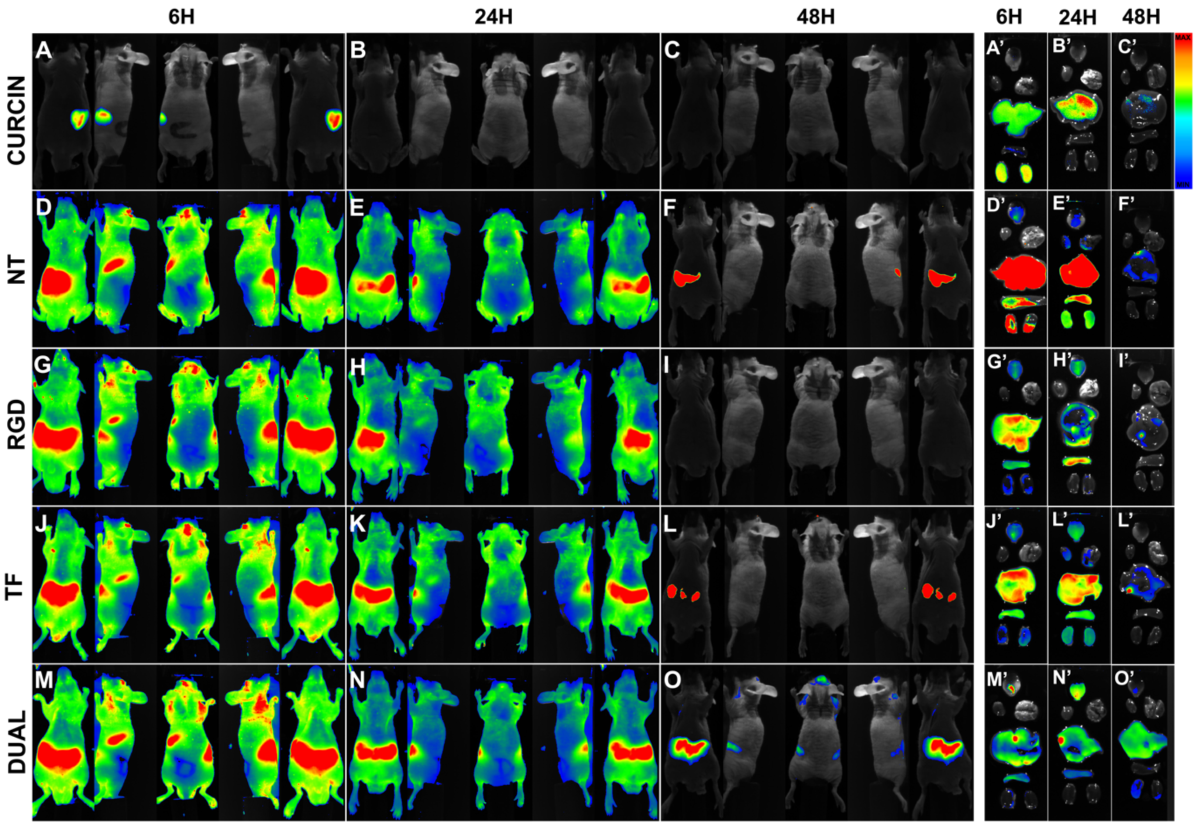

Figure S10. ***In vivo*** **biodistribution and tumor-targeting of HSLNs in gliosarcoma-bearing mice (A-C)** Whole-body fluorescence imaging of mice treated with ICG-bound free curcin at 6 h **(A)**, 24 h **(B)**, and 48 h **(C)**. Signal was limited primarily to hepatic and renal regions. **(D-O)** Whole-body biodistribution of NT **(D-F)**, RGD **(G-I)**, TF **(J-L)**, and Dual **(M-O)** HSLN–CdSe at 6 h, 24 h, and 48 h post-injection. Rapid systemic distribution was observed across all groups at 6 h, followed by progressive clearance through 48 h. Among all variants, Dual HSLNs exhibited sustained fluorescence signal in the cranial region up to 48 h, indicative of extended tumor retention and superior BBB translocation. **(A'-O')** *Ex vivo* organ imaging at corresponding timepoints. All formulations, including curcin localized predominantly to liver and kidney, with varying degrees of accumulation. HSLNs (RGD, TF, Dual) fluorescence was seen in the excised brain, especially near the tumor regions. Notably, Dual HSLNs **(M'-O')** showed consistent accumulation within the gliosarcoma-implanted hemisphere, with minimal off-target deposition. These biodistribution patterns confirm that Dual HSLNs effectively penetrate the BBB, achieve high tumor-to-brain selectivity, and demonstrate prolonged systemic circulation. Among all groups, Dual HSLNs showed the most persistent and localized signal within the tumor site. These findings were supported by ICP-MS quantification **(Figure 2R–U)**, which revealed minimal cadmium signal in normal brain, maximum uptake in tumor tissue, and a ~7-fold increase in tumor accumulation versus NT and ~2-fold over single-ligand systems.

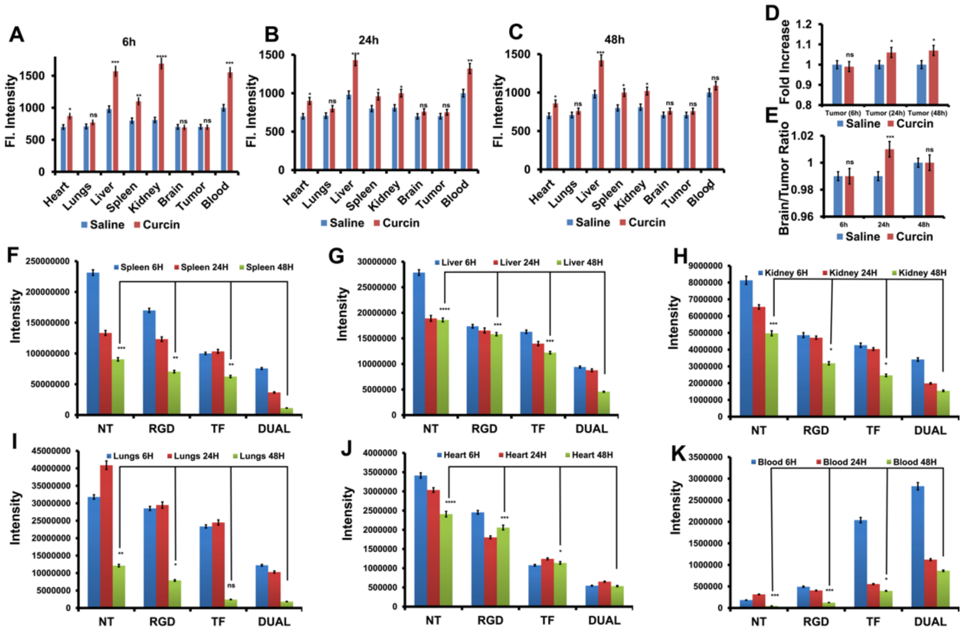

Figure S11. Quantitative fluorescence biodistribution analysis in gliosarcoma-bearing mice following nanoparticle or free curcin administration (A-C). Quantified fluorescence intensity in major organs at 6 h **(A)**, 24 h **(B)**, and 48 h **(C)** post-administration of ICG-bound free curcin or saline (control). Free curcin exhibited statistically significant accumulation in liver, spleen and kidney at all time points compared to saline, with no significant difference in brain and tumor, indicating minimal CNS penetration. **(D)** Fold increase in tumor-localized fluorescence intensity (tumor targeting efficiency) revealed slight, time-dependent enhancement with curcin versus control. **(E)** Brain-to-tumor signal ratio at 6, 24, and 48 h indicated a slight increase in tumor accumulation. All comparisons are presented as mean ± SD (n = 3), with statistical significance determined by one-way ANOVA and Tukey's post hoc test. ns = not significant; $p < 0.05$ (*), $p < 0.01$ (**), $p < 0.001$ (***), $p < 0.0001$ (****). **(F-K)** ICP-MS quantification of organ-specific clearance profiles over 6, 24, and 48 h in animals treated with NT, RGD, TF, and Dual HSLN–CdSe variants. Spleen **(F)**, liver **(G)**, kidney **(H)**, lungs **(I)**, heart **(J)**, and blood **(K)** samples were analyzed for signal intensity. NT HSLNs exhibited highest fluorescence in reticuloendothelial organs (liver, spleen, lungs), consistent with nonspecific RES clearance. RGD and TF variants showed moderate signal reduction over time. Dual HSLNs demonstrated the most rapid systemic clearance and the lowest non-tumor signal at 48 h, highlighting the reduced off-target burden. Blood fluorescence levels for Dual HSLNs were sustained at 6 h but declined significantly by 24–48 h, supporting favorable pharmacokinetics for tumor-targeted delivery with minimal prolonged systemic exposure. These results provide quantitative confirmation of biodistribution trends observed *in vivo* and *ex vivo* **(Figure S10)**, reinforcing the tumor selectivity and reduced systemic retention of Dual HSLNs. Together, these data support the enhanced circulation kinetics and safety profile of the dual-targeted nanoformulation. **Note**: In the Y-axis, scales vary between subplots to allow accurate resolution of organ-specific signal trends. Uniform scaling was avoided due to significant inter-organ differences in fluorescence intensity, which would otherwise compress lower-intensity signals and obscure biologically meaningful differences—especially for low-uptake tissues such as brain and tumor. This representation allows a fair and interpretable comparison of biodistribution kinetics across both high- and low-accumulation organs.

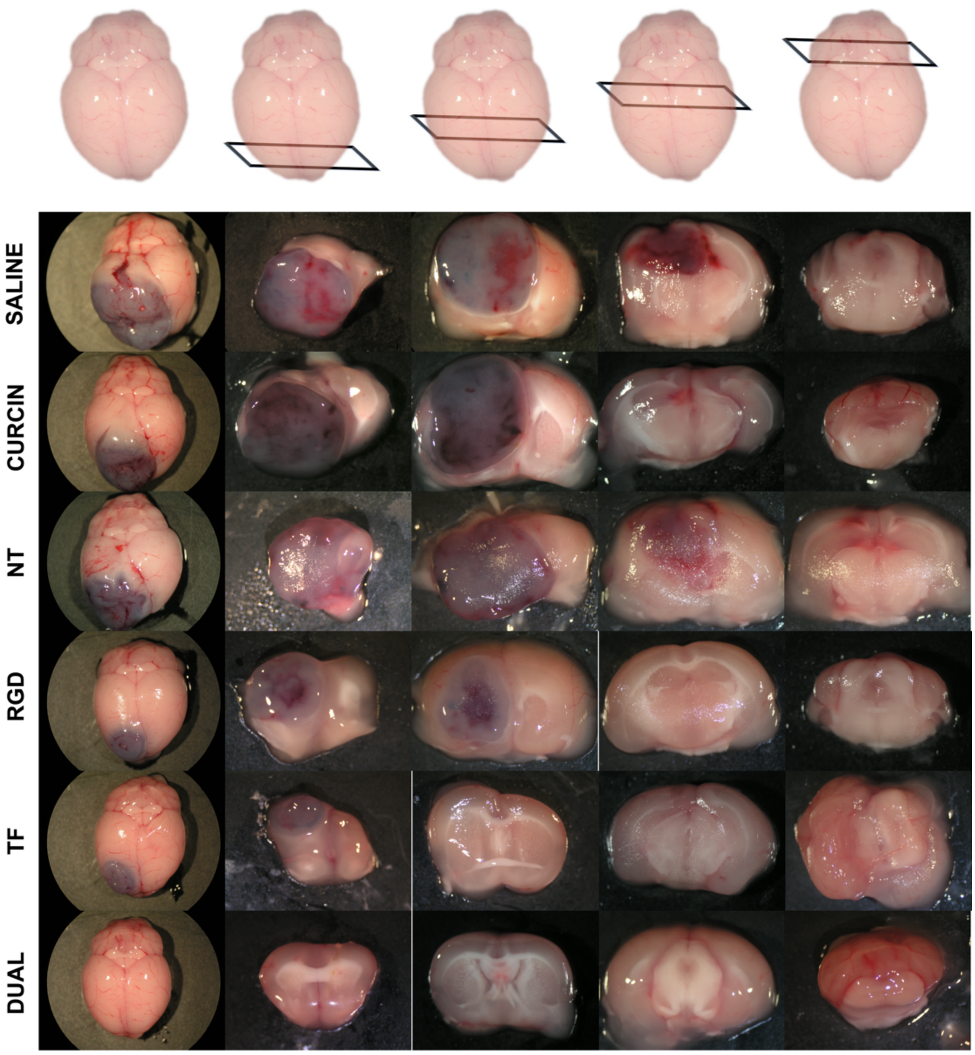

Figure S12. Evaluation of BBB integrity and tumor invasiveness using Evans Blue dye extravasation. Coronal sections of excised mouse brains from each treatment group (Saline, Curcin, NT-HSLN, RGD-HSLN, TF-HSLN, and Dual-HSLN) were collected postmortem following intravenous injection of 2% Evans Blue dye. The top schematic indicates the anatomical planes at which sections were obtained for visual comparison across multiple depths of the brain. In saline-, free curcin-, NT-, RGD-, and TF-treated animals, prominent Evans Blue staining was observed, particularly in the tumor-implanted hemisphere, indicating substantial BBB disruption and high vascular permeability associated with gliosarcoma progression. In contrast, brains from the Dual HSLN group showed markedly reduced or absent dye infiltration, consistent with preserved BBB integrity and limited peritumoral leakage.
These results demonstrate that only Dual HSLNs effectively restrict vascular remodeling and pathological BBB breakdown within the gliosarcoma microenvironment. This observation supports findings from integrisense imaging **(Figure 4C)**, in which tumor-associated vasculature was largely absent in the Dual group, and further correlates with the reduced angiogenic and proinflammatory marker expression seen in immunohistochemistry **(Figure 4E-I)** and proteomic profiling **(Figure 4J; Table S2)**. Evans Blue retention patterns in this figure serve as a functional readout of nanoparticle-mediated tumor vascular normalization, further distinguishing Dual HSLNs as uniquely capable of achieving selective therapeutic effects while maintaining the integrity of surrounding healthy brain vasculature.

Table S2. Protein expression analysis between test groups: Their name, gene annotation and functions in tumorigenesis.

Protein	Encoding Gene	Function
Angiogenin	ANG	• Upregulated and associated with poor prognosis in human cancers and strongly correlated with an invasive cancer phenotype.<br>• Known to induce cellular survival, proliferation, endothelial tube formation and xenograft angiogenesis and growth.<br>• Novel mechanistic investigations revealed that ANG expression stimulated matrix metallopeptidase-2 (MMP2) expression through the phosphorylation of ERK1/2.<br>• Inhibition of ANG is known to result in the diminution of xenograft tumoral growth through the inhibition of angiogenesis.
Pro-epidermal Growth Factor	EGF	• EGF-induced signaling has often been associated with tumor invasion and metastasis and has been correlated with an advanced tumor stage and a poor clinical prognosis.<br>• It has been reported that EGF promotes tumor cell motility and invasion.<br>• EGF-induced three-dimensional cell-cell or cell-ECM interactions *in vivo* are also important regulators of tumor cell proliferation.
Basic Fibroblast Growth Factor (bFGF)	FGF2	• FGF2 plays a significant part in regulating GBM.<br>• FGF2 is commonly included as a supplement in media used to culture GSCs *in vitro*.<br>• FGF can act synergistically with vascular endothelial growth factor (VEGF) to amplify tumor angiogenesis<br>• FGF has the potential to overcome chemotherapy resistance
Matrix metalloproteinase-9	MMP9	• MMPs are a family of zinc-dependent endopeptidases<br>• Mediate many of the changes in the microenvironment during tumor progression.<br>• Regulate variety of physiological processes and signaling events<br>• Represent key players in the molecular communication between tumor and stroma.
Platelet-derived growth factor subunit B	PDGFB	• PDGF-B is one of the most abundant growth factors in the tumor microenvironment secreted by tumor cells.<br>• PDGF-B has been implicated in promoting proliferation, survival, and CAF migration in these desmoplastic tumors

Vascular endothelial growth factor A	VEGFA	• Growth factor active in angiogenesis, vasculogenesis and endothelial cell growth.<br>• Induces endothelial cell proliferation, promotes cell migration, inhibits apoptosis and induces permeabilization of blood vessels
Vascular endothelial growth factor C	VEGFC	• Growth factor active in angiogenesis, and endothelial cell growth<br>• Stimulates proliferation and migration of endothelia and also has effects on the permeability of blood vessels.
Urokinase-type plasminogen activator	PLAU	• Specifically cleaves the zymogen plasminogen to form the active enzyme plasmin
Granulocyte-macrophage colony-stimulating factor	CSF2	• Cytokine that stimulates the growth and differentiation of hematopoietic precursor cells from various lineages, including granulocytes, macrophages, eosinophils and erythrocytes
Plasminogen activator inhibitor 1	SERPINE 1	• Serine protease inhibitor.<br>• This inhibitor acts as 'bait' for tissue plasminogen activator, urokinase, protein C and matriptase-3/TMPRSS7.<br>• Its rapid interaction with PLAT may function as a major control point in the regulation of fibrinolysis
C-C motif chemokine 2	CCL2	• Chemotactic factor that attracts monocytes and basophils but not neutrophils or eosinophils.<br>• Augments monocyte anti-tumor activity
Endoglin	ENG	• Vascular endothelium glycoprotein that plays an important role in the regulation of angiogenesis
Platelet-derived growth factor subunit A	PDGFA	• Growth factor that plays an essential role in the regulation of embryonic development, cell proliferation, cell migration, survival and chemotaxis
Metalloproteinase inhibitor 1	TIMP1	• Functions as a growth factor that regulates cell differentiation, migration and cell death and activates cellular signaling

Table S3. Proteins of interest that occur repeatedly among functional partner links. As these interactions are critical to many important cellular functions and their dysregulation is causal of disease, the modulation of these binding events has emerged as a leading yet difficult therapeutic arena. Protein-protein interaction networks, relevant to this study are of fundamental importance as the tumor-promoting function of several aberrantly expressed proteins in the cancerous state is directly resultant of their ability to interact with a protein-binding partner.

**Protein**	**Occurring Protein-Protein Interaction Links**
EGF	PDGF, TIMP1, VEGFA, VEGFC
MMP9	PLAU, TIMP1
PDGFB	PDGFA, VEGFC
PLAU	SERPINE1, MMP9
SERPINE1	PLAU
TIMP1	MMP9
VEGFA	TIMP1, ANG, EGF, MMP9, PDGFB

Table S4. Co-expressing proteins along with the protein of interest and their co-expression score. The resulting co-expression networks are used to identify genes functionally related or controlled by the same transcriptional regulatory system.

Protein	Co-expressing Protein	Co-expression Score
ANG	RNASE4 (Ribonuclease)	0.856
CCL2	IL6 (Cytokine)<br>CCR1 (Cytokine)	0.233<br>0.086
PLAU	SERPINE1<br>PLAUR (Receptor for uPA)	0.102<br>0.225
SERPINE1	PLAU	0.102
TIMP1	THBS1 (Glycoprotein)<br>MMP14 (Matrix metalloproteinases)<br>MMP2 (Matrix metalloproteinases)	0.088<br>0.072<br>0.091
VEGFC	FN1 (Fibronectin)<br>THBS1	0.091<br>0.088

Table S5. Neurological and behavioral outcomes associated with tumor-induced disruption of specific brain regions; This table summarizes the observable physiological, behavioral, and neurological deficits observed in this study, resulting from gliosarcoma progression and tumor-induced damage to key brain areas. Clinical signs such as seizures, paralysis, sensory loss, impaired thermoregulation, and feeding behavior are mapped to their underlying neuropathological basis. Numerical labels in parentheses correspond to specific anatomical regions detailed in **Table S6**.

**Seizure**	Electrical Imbalance in Brain **(12, 18, 24, 31, 42)**
**Paralysis**	Loss of Muscle Function **(1, 7, 13, 28, 40, 41)**
**Pale Body**	Reduced Thickness and Density of Blood Vessels
**Spontaneous Shock and Death**	Heart Attack/Brain Dead **(14)**
**Weak and Feeble**	Loss of Appetite and Thirst
**Cold Body**	Impaired Heat-gain to Heat-loss crosstalk **(48)**
**Blood Clots On Brain Surface**	Leaky and Aberrant Blood Vessels; Compromised Permeability; Excessive Clot Behavior
**Tumor Tissue Fused to Skull**	Cell-Cell Interaction Compromised; Outgrowth of Tumor
**No Consumption of Water**	Visceral Area Damage **(16)**
**No Food Intake**	Visceral Area Damage **(16)**
**Biased Rotational Behavior**	Navigation Impairment/Spatial Reasoning Deficit **(7, 13, 28)**
**Decreased Alertness or Escape Behavior**	Loss of Eyesight and Smell **(4, 6, 15, 19, 26, 29, 34, 35, 53, 56)**
**No Sense of Food**	Loss of Smell **(4, 19, 20, 57)**

Table S6. Brain Regions Affected by Tumor Expansion and Their Functional Roles: This table provides a comprehensive mapping of brain structures compromised by gliosarcoma progression, along with their corresponding neurophysiological functions. Brain areas are grouped into major domains including the cerebral cortex, fiber tracts, midbrain, hindbrain, cerebral nuclei, and hypothalamic-thalamic regions. The numerical identifiers are cross-referenced in **Table S5** to contextualize specific behavioral and physiological deficits observed in tumor-bearing subjects.

**Cerebral Cortex**	Primary and Secondary Motor Area	Control of movements **(1)**
	Prelimbic Area	Emotional response **(2)**
	Orbital Area	Cognitive processing **(3)**
	Olfactory area	Sensation of smell **(4)**
	Retrosplenial Area	Perception and memory **(5)**
	Primary, Postero-Lateral, Lateral Visual Area	Process visual information **(6)**
	Posterior Parietal Association Area	Planned movements, spatial reasoning and attention **(7)**
	Temporal Association Area	Recognition and identification of stimuli **(8)**
	Ectorhinal Area	Memory and navigation **(9)**
	Perihinal Area	Sensory information processing **(10)**
	Pre, Post and Subiculum Area	Working memory **(11)**
	Dentate Gyrus	Receives excitatory neurons of learning, memory and spatial coding **(12)**
	Entorhinal Area	Memory, navigation and spatial memories **(13)**
	Anterior Cingulate Area	Autonomic functions (regulates BP and heart rate), also involved in decision making, impulse control and emotion **(14)**
	Primary and Supplemental Somatosensory Cortex	Sensory reception area **(15)**
	Visceral Area	Visceral sensation, hunger, thirst, change in body temperature **(16)**
	Gustatory Cortex	Perception of taste **(17)**
	Claustrum	Bridges communication between brain hemispheres **(18)**
	Piriform Cortex	Perception of smell **(19)**

	Ventral Auditory Area	Processes auditory information **(20)**
	Field CA1, CA2, CA3	Structural unit of hippocampal formation **(21)**

**Fiber Tracts**	Olfactory Nerve	Transmit nerve impulse about odors to CNS **(22)**
	Cingulum	Cognitive function and emotion **(23)**
	Corpus Callosum	Neural impulse transmission **(24)**
	Anterior Commisure	Pain and pain sensation, smell and chemoreception **(25)**
	Lateral Olfactory Tract	Sense of smell **(26)**
	Fornix	Carries signal from hippocampus to mammillary bodies **(27)**
	Corticospinal tract	Voluntary motor control of body and limbs **(28)**
	Optic tract	Visual system in brain **(29)**
	Mammillary tract	Recollective memory **(30)**
	Superior Cerebellar Peduncle	Cerebellum to midbrain neuron signal transmission **(31)**
	Lateral and Medial Lemniscus	Sound impulse and somatosensation **(32)**
	Cerebral Peduncle	Guards spinal tracts **(33)**
	Tectospinal Pathway	Coordinates head and eye movement **(34)**

**Midbrain**	Superior Colliculus	Respond to visual stimuli, motor related **(35)**
	Periaqueductal Grey	Pain modulation **(36)**
	Midbrain Reticular Nucleus	Behavioral arousal and consciousness **(37)**

**Hindbrain**	Superior Central Nucleus	Behavioral changes **(38)**
	Tegmental Reticular Nucleus	Reflexes and posture **(39)**
	Pontine Grey	Motor skills **(40)**

**Cerebral Nuclei Region**	Caudaputamen	Regulate movement and influence various types of learning **(41)**
	Lateral Septal Complex	Reciprocal connections from the olfactory bulb, hippocampus,

		hypothalamus, midbrain and thalamus **(42)**
	Nucleus accumbens	Perception of pleasant emotional scenes, rewarding and reinforcing stimuli **(43)**
	Pallidum	Regulation of behavior and emotions **(44)**
	Olfactory Tubercle	Multisensory processes, reward and arousal **(45)**
	Amygdalar Area	Memory, decision making and emotional reactions **(46)**
	Amygdalar Nucleus	Receive and process pain **(47)**

**Thalamus and Hypothalamus**	Preoptic Region	Thermoregulation, propagates stimuli to heat-losing or heat-promoting centers **(48)**
	Vascular Organ of Lamina Terminalis	Sensory circumventricular organ that serve as osmoreceptors **(49)**
	Epithalamus	Secretion of melatonin and hormones, also regulates motor pathways and emotions (50)
	Polymodal Association Cortex	Knowledge processing and memory **(51)**
	Lateral Habenula	Stimuli associated with unpleasant events **(52)**
	Anterior Group of Dorsal Thalamus	Modulation of alertness, involved in learning **(53)**
	Ventral Posterior Complex	Involved in touch, body position, pain, temperature and taste **(54)**
	Hypothalamic Medial Zone	Regulates release of gonadotrophic hormones **(55)**
	Lateral Hypothalamic Area	Feeding behavior and wakefulness **(56)**
	Ventromedial Nucleus	Involved in satiety and neuroendocrine control **(57)**
	Arcuate Nuclei	Feeding and activates release of growth hormones **(58)**

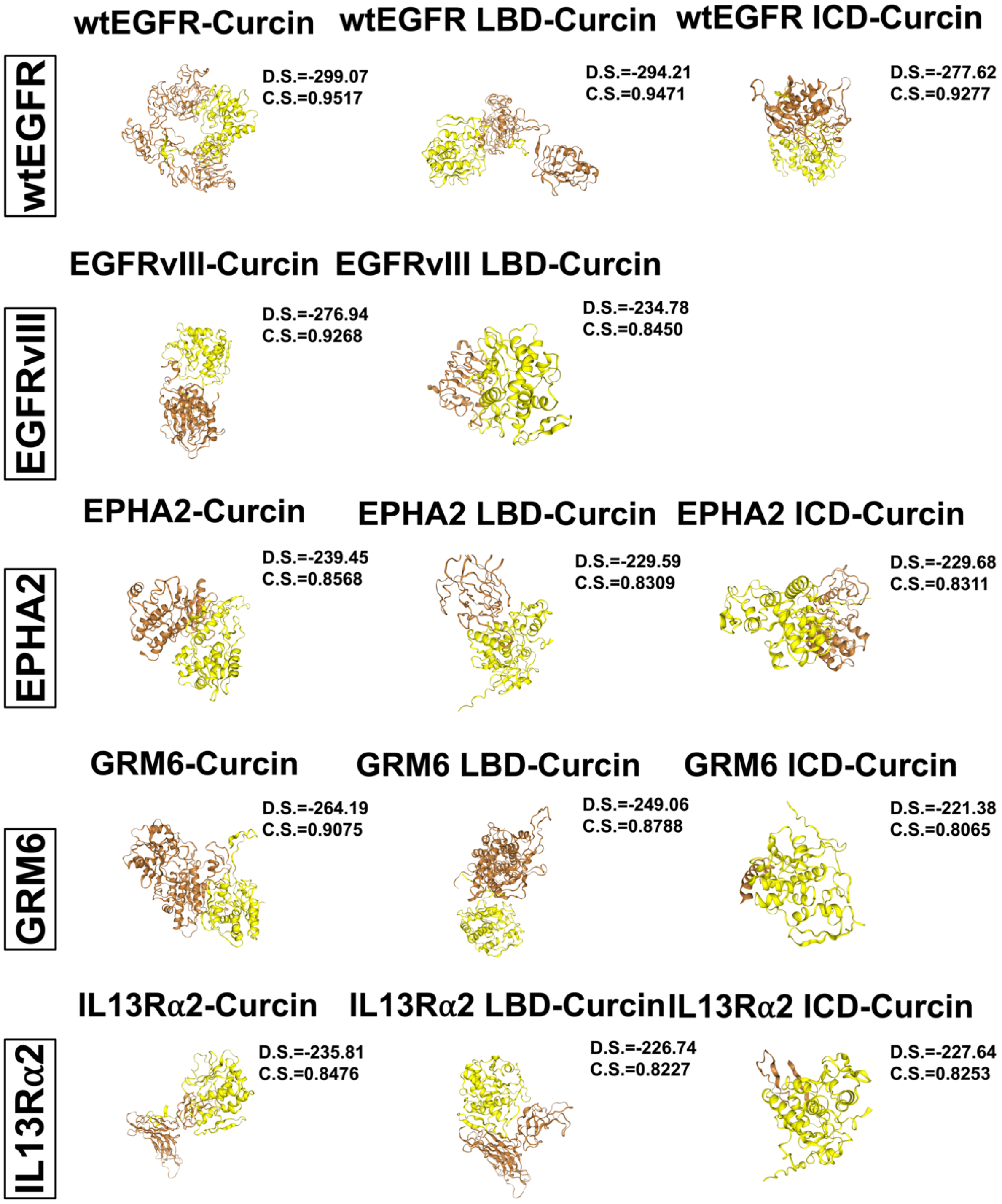

Figure S13. Molecular docking analysis of curcin with gliosarcoma-associated receptors. Computational docking was performed to evaluate the interaction of curcin with four gliosarcoma-associated cell surface receptors: wild-type EGFR, EGFRvIII, EphA2, IL-13Rα2, and metabotropic glutamate receptor 6 (mGluR6/GRM6). For each receptor, curcin was docked against the full-length structure, ligand-binding domain (LBD), and intracellular domain (ICD), where applicable. Protein structures were obtained from the RCSB PDB or AlphaFold, and docking simulations were executed using the HDOCK server with hybrid template-based and ab initio modeling. Protein–ligand complexes were visualized, and corresponding docking

scores (D.S.) and confidence scores (C.S.) are shown. Curcin demonstrated strong binding affinity to all tested receptor domains, with docking scores ranging from –221 to –299 (a.u.) and confidence scores exceeding 0.80 in all cases. Binding to both LBDs and ICDs suggests a potential bifunctional inhibitory mechanism, whereby curcin may competitively inhibit extracellular ligand binding and simultaneously interfere with intracellular dimerization, autophosphorylation, or downstream signaling events. Among the receptor targets, the strongest binding was observed for wild-type EGFR (D.S. = –299.07, C.S. = –0.9517), followed by GRM6 and EGFRvIII, both of which are implicated in gliosarcoma resistance and recurrence. Notably, docking to the LBD of EphA2 and the ICD of IL-13Rα2 also produced favorable scores, supporting a multi-receptor engagement model. These findings are consistent with curcin's *in vivo* effects observed in Dual HSLN-treated mice, which displayed enhanced tumor regression, vascular remodeling, and prolonged survival, potentially reflecting additive inhibition of multiple receptor-driven pathways. Compared to traditional small-molecule inhibitors that often target only one domain or receptor, curcin's multi-site interaction profile highlights its polypharmacological potential, especially when delivered via dual-ligand-targeted nanoparticles. These results support its classification as a bifunctional anti-gliosarcoma agent and reinforce its therapeutic relevance in receptor-heterogeneous tumor microenvironments.
